# Novel reusable animal model for comparative evaluation of *in vivo* growth and protein-expression of *Escherichia coli* O157 strains in the bovine rumen

**DOI:** 10.1371/journal.pone.0268645

**Published:** 2022-05-26

**Authors:** Indira T. Kudva, Julian Trachsel, Erika N. Biernbaum, Thomas Casey

**Affiliations:** 1 Food Safety and Enteric Pathogens Research Unit, National Animal Disease Center, Agricultural Research Service, U.S. Department of Agriculture, Ames, Iowa; 2 Oak Ridge Institute for Science and Education (ORISE), ARS Research Participation Program, Oak Ridge, Tennessee; INRAE Centre Val de Loire: Institut National de Recherche pour l’Agriculture l’Alimentation et l’Environnement Centre Val de Loire, FRANCE

## Abstract

Previously, we had demonstrated that *Escherichia coli* O157:H7 (O157) strain 86–24 expresses proteins involved in survival rather than virulence *in vitro* in rumen fluid from dairy cattle limit fed a maintenance diet. Here, we verified if this observation would be true for different O157 strains grown *in vitro* in rumen fluid from, and *in vivo* in the rumen of, animals on contrasting maintenance (high fiber) and lactation (high energy-protein) diets usually limit fed to dairy cattle. For the *in vivo* studies, an economical, novel, reusable and non-terminal rumen-fistulated animal model permitting simultaneous evaluation of multiple bacterial strains in the bovine rumen was developed. All experiments were conducted in duplicate using different animals to account for host-related variations. The O157 strains included, 86–24, EDL933 and the super shed SS-17. *E*. *coli* Nal^R^ (#5735), derived from a bovine intestinal commensal *E*. *coli*, was included as a control. As expected, diet influenced ruminal pH and volatile fatty acid (VFA) composition. The pH ranged from 6.2–7.0 and total VFA concentrations from 109–141 μM/ml, in animals fed the maintenance diet. In comparison, animals fed the lactation diet had a ruminal pH ranging between 5.18–6.0, and total VFA of 125–219 μM/ml. Strain dependent differences in O157 recovery from the rumen fluid of cattle fed either diet was observed, both *in vitro* and *in vivo*, with O157 strains 86–24 and EDL933 demonstrating similar survival patterns. Analysis of the O157 proteomes expressed in the rumen fluid/rumen verified previous observations of adaptive responses. Any difference in the adaptive response was mainly influenced by the animal’s diet and growth conditions (*in vitro* and *in vivo*) and not the O157 strain. These new insights into the O157 responses could help formulate modalities to control O157 across strains in cattle at all stages of husbandry.

**Importance of this study**

This study demonstrates that O157, irrespective of strain, have similar survival and protein expression patterns in rumen fluid with variations being influenced primarily by rumen fluid composition and the *in vitro* or *in vivo* environment. Hence, evaluating microbes within the host is critical when seeking to understand colonization dynamics and identifying targets for control strategies. Most importantly, the method presented here for conducting such *in vivo* studies in the rumen will permit reuse of the animal and allow for multiple evaluations.

## Introduction

Shiga toxin-producing *Escherichia coli* (STEC) is the third leading cause of foodborne illness after *Campylobacter* and *Salmonella*, implicated in 265,000 illnesses annually in the US [1,2]. Globally, STEC infections are reported to cause approximately 2.8 million acute illnesses with 230 deaths annually [3]. *Escherichia coli* O157:H7 (O157) was the first STEC serotype to be associated with diarrhea, bloody diarrhea or hemorrhagic colitis and hemolytic uremic syndrome (HUS) in humans [4–7] and has since caused frequent outbreaks in the US [2,8]. STEC such as O157 can infect humans in low doses (~10 viable bacteria) [5,9–11] and are acquired through the fecal-oral route following ingestion of bacteria-contaminated food or water, or after contact with infected animals and humans [5,9,12,13]. No specific therapies are available for treating STEC infections in humans. Cattle are considered the primary STEC reservoirs as most outbreaks are directly or indirectly associated with cattle [14,15]. Cattle remain asymptomatic [16], although STEC can be isolated from various gastrointestinal tract sites and tend to persist at the rectoanal junction (RAJ) [17–19]. Cattle shed STEC in a seasonal pattern, with increased shedding in warmer months and decreased shedding in winter [15]. Average duration of bovine O157 carriage is 30 days, although colonization of up to 1 year has been reported [20–22]. The typical shedding rate of O157:H7 from the RAJ is 10–100 colony forming units per gram (CFU/g) of feces [23]. Animals shedding greater than 10^4^ CFU/g feces, termed “super-shedders” contribute to herd prevalence of STEC [24,25].

Studies evaluating colonization sites within the bovine gastrointestinal tract (GIT) have invariably recovered some O157 from the rumen even if in relatively low numbers compared to other GIT compartments [19,26]. Considering the challenging environment in the rumen with feed, digesta, diverse microbiota, and various chemicals, it is of interest to understand mechanisms of bacterial survival and chemical sensing in this GIT compartment that enables downstream colonization of other intestinal sites [18,19,27–31]. Chemical sensing studies examining AHL (acyl-homoserine lactones) sensing have been conducted to either establish the role of SdiA, an AHL receptor, in the bacterial-bovine commensal relationship [27,32] or to examine the effect of AHLs on STEC colonization after consumption of various diets [33]. For instance, rumen cannulated heifers fed a grain diet [32] or steers fed a grain or hay (forage) diet [33] were inoculated with STEC and changes in rumen AHLs were analyzed using thin-layer chromatography and correlated with ruminal bacteria recovered. Studies like these could provide insights into reducing STEC shedding by manipulating specific chemical sensing pathways and by feeding cattle a diet less conducive to STEC colonization. Probiotic, microbiome, and proteomic analyses have also been conducted using rumen fluid. Bertin *et al*. explored the use of *Lactobacillus reuteri* strains as potential probiotics for finishing beef cattle prior to slaughter to reduce ruminal STEC concentrations [34]. In that study, glycerol-supplemented rumen fluid from cannulated Holstein cattle was co-inoculated with *L*. *reuteri* and STEC strains FCH6 and EDL933 under anaerobiosis *in vitro*, to demonstrate STEC suppression by the putative probiotic bacteria [34]. A similar study by Zhou *et al*. assessed the impacts of Tasco, an *Ascophyllum nodosum* extract marketed by Acadian Seaplants Ltd., Dartmouth, NS, Canada, on the ruminal microbiome and STEC in rams to serve as a feed additive to reduce fecal STEC shedding [31]. Tasco decreased *E*. *coli* populations in the rumen 10-fold and reduced O45, O103, O111, and O121 in the feces [31]. Additionally, rumen fluid has been used as a means of analyzing bacterial survival. One such study by Free *et al*. compared *in vitro* survival of O157 and non-O157 STEC in rumen fluid medium and observed significant decline in viable counts for serotypes O103:K:H8 and O26:H11 post-24 hr incubation [35].

In our previous study, we cultured O157 strain 86–24 *in vitro* in different preparations of rumen fluid and LB media and observed that O157 primarily expresses proteins involved in bacterial adaptation and not virulence in rumen fluid [28]. However, most studies including ours, evaluating STEC growth in bovine rumen fluid and making inferences were either conducted *in vitro*, or in terminal *in vivo* experiments with limited bacterial recovery from the rumen. Hence, we sought to develop a method that would permit non-terminal *in vivo* studies in rumen cannulated animals with ensured recovery of bacterial inoculum. Once the method was standardized, we evaluated three different O157 strains in the rumen of cows on contrasting diets usually fed to dairy cattle and compared the *in vivo* bacterial profiles to that observed *in vitro* in the same rumen fluid to determine differences in O157 survival and adaptive responses.

## Materials and methods

### Bacterial strains

The four different *E*. *coli* strains used in this study included, **(i)** *E*. *coli* Nal^R^ (NADC stock #5735; *stx1*-, *stx2*-, *eae*-), a non-pathogenic, nalidixic-resistant derivative of a bovine commensal *E*. *coli*, **(ii)** O157 strain 86–24 (*stx2*+, *eae*+), a wild-type, human, O157:H7 isolate from an outbreak in Washington State [36,37], **(iii)** O157 strain EDL933 (ATCC®43895™; *stx1*+, *stx2*+, *eae*+), a wild-type, human, O157:H7 isolate associated with the 1982 multi-state outbreak involving contaminated hamburgers (6), and **(iv)** O157 strain SS17 (*stx2*+, *eae*+), a bovine, wild-type, super-shed O157 [38].

### Rumen fistulated cattle

All animal experiments were done using protocols approved by the Institutional Animal Care and Use Committee at the National Animal Disease Center (NADC, Ames, IA). A total of six rumen-fistulated Holstein heifers/cows between 1 to 4 years of age and routinely used as rumen fluid ‘donors’ at the NADC were utilized for this project. One animal (animal #A) was part of the pilot study standardizing the ruminal-challenge method with *E*. *coli* Nal^R^ (#5735) and five (animals #A2, B, C, D, E) were used in the test experiments evaluating the three O157 strains with *E*. *coli* Nal^R^ (#5735) as a control. Feed intake and body temperatures were monitored on a daily basis. Animals were housed in the field barn until ready for ruminal-challenge with O157 strains when they were moved in to the BSL-2 containment barns at the NADC. At the end of each study, following confirmation of their non-O157 shedding status, all animals were returned to the field barn. The animals were limit fed a total mixed ration maintenance diet (M diet) for the first half of the study lasting for 2 weeks following which the diet was gradually switched to the lactation diet (L diet) over 2 weeks; the L diet was continued into the second half of the study lasting for another 2 weeks. Water was provided ad libitum throughout. The maintenance diet is usually formulated such that feed energy intake will result in no net loss or gain of energy in the cow’s body thereby maintaining overall weight [39,40]. On the other hand, a lactation diet is designed to meet the high energy needs of the cow during milk production [39,40]. All dietary compositions were monitored by an animal nutritionist on site for our study to closely match the defined requirements. The M diet consisting of 25% grass hay, 65% corn silage, and 10% Steakmaker 40–20 (product composition label is shown in S1 Fig. Land O’Lakes, Inc., Arden Hills, Mn.) was limit fed at ~19 lbs/head with access to pasture or at ~30 lbs/head without pasture. The L diet was limit fed at ~99–100 lbs/head and included 2.5% grounded corn, 62% corn silage, 2.5% soybean meal, 12% legume hay and 21% lactation premix (product composition label is shown in S1 Fig).

### Bacterial culture

#### (i) Inoculum preparation

Overnight bacterial cultures grown in Luria-Bertani (LB) broth, with or without nalidixic acid (100 μg/ml; LB-Nal), at 39°C with aeration, were used to set up log-phase sub-cultures in 50 ml LB/LB-Nal broth, under the same growth conditions. Bacteria harvested from the log-phase cultures at an OD_600_ 0.5–0.6, washed and re-suspended in sterile 0.9% saline, were used to charge dialysis tubings or cartridges as described below under “*In vitro and in vivo evaluation of bacterial strains*”. All O157 cultures were confirmed serologically using latex agglutination kits (*E*. *coli* O157 latex, Oxoid Diagnostic Reagents, Oxoid Ltd., Hampshire, United Kingdom) and plated for viable counts as described below.

#### (ii) Isolation of bacteria from dialysis tubings/cartridges

Serial dilutions of bacterial suspensions, aspirated from the dialysis tubings/cartridges post-incubation, were prepared using sterile saline and plated on to LB-Nal and/or sorbitol MacConkey (BD Biosciences) with 4-methylumbelliferyl-β-d-glucuronide (100 mg/liter; Sigma; SMAC-MUG) agar to determine viable colony counts in CFU/ml. Bacteria adhering to the tubing or cartridge walls were scrapped off, using sterile inoculum loops, into the suspension within before aspirating the same. Bacterial suspensions and colonies were evaluated for the O157 serotype using latex agglutination kits (*E*. *coli* O157 latex, Oxoid).

#### (iii) Rumen fluid and fecal sample testing for O157

To determine the presence of any O157 in the animal, rumen fluid and fecal samples were cultured, over two weeks, both at the beginning and end of study. In an unrelated study, direct inoculation of the rumen with 8 ml of 10^9^ cfu/ml O157 strain 86–24 resulted in the recovery of the bacteria in cattle by selective-enrichment from feces within 24 h of inoculation and from the rumen fluid after a week (data not shown). Hence, a two week period was chosen to monitor any possible contamination of the animals used in this study. Previously standardized non-enrichment and selective-enrichment culture protocols were used with modifications [41–43]. Briefly,10 ml rumen fluid or 10-g fecal sample was added to 50 ml Trypticase soy broth (BD Bioscience, San Jose, Ca.) supplemented with cefixime (50 μg/liter; U.S. Pharmacopeia. Washington D.C), potassium tellurite (2.5 mg/liter; Sigma-Aldrich Corp., St. Louis, Mo.), and vancomycin (40 mg/liter; Alfa Aesar, Haverhill, Ma.) (TSB-CTV) or LB-Nal broth and mixed well. Serial dilutions of each sample were prepared with sterile 0.9% saline both before and after overnight incubation of the TSB-CTV or LB-Nal suspension at 37°C with aeration. The dilutions prepared before incubation (non-enrichment cultures) and after overnight incubation (selective-enrichment cultures) were spread plated onto SMAC-MUG and LB-Nal plates. SMAC-MUG plates were read after overnight incubation at 37°C and colonies that did not ferment sorbitol or utilize 4-methylumbelliferyl-β-d-glucuronide (non-fluorescent under UV light) were further evaluated to be O157 serologically.

### Rumen fluid preparation and analysis

Rumen fluid samples collected from rumen-fistulated cows, fed the M diet (MRF) or the L diet (LRF), were used for culture and to set up the *in vitro* experiments for this study. Up to five liters of either rumen fluid was collected 2–3 hr post-feeding to allow for rumination to occur, at each sampling [44,45]. As previously described, the rumen fluid was strained through cheesecloth to remove large feed particles and poured into collection flasks [28]. Following recording of the pH and freezing of aliquots for volatile fatty acid (VFA) analysis, the remaining strained rumen fluid was aliquoted into flasks for *in vitro* studies as described below. The pH of the rumen fluid was determined using pH paper (pH range 5.0–8.0; Micro Essential Laboratory Inc., Brooklyn, NY) and VFA concentrations determined by capillary gas chromatography, as described previously [46,47], on an Agilent 6890 N gas chromatograph (Agilent Technologies, Inc., Santa Clara, CA). Three technical replicates were used per rumen fluid sample to determine VFA concentrations. The panel of substrates evaluated to determine total VFA included various by-products of carbohydrate and protein fermentation such as, acetate, propionate, butyrate, isobutyrate, formate, lactate, valerate, isovalerate, caproate, oxalate, phenylacetate, succinate and fumarate [39,40,48]. However, three VFA concentrations, acetate, propionate, and butyrate, were specifically studied as these influence the growth and energy dynamics of the bovine host [39,40,48]. The rumen fluid pH and VFA were recorded before and after introduction of O157 strains at the end of each incubation, in both *in vitro* and *in vivo* studies. Intermittent sampling was avoided to prevent any inadvertent influence of repeated exposure of rumen fluid/rumen to external environment.

### *In vitro* and *in vivo* evaluation of bacterial strains

All experiments were done in duplicate and followed the plan as shown in S2 Fig. Since the overall goal was to determine the influence of diet on bacterial gene expression in the rumen fluid, steps were taken to minimize any other host-related effects. This was done by mixing rumen fluid collected from different animals for the *in vitro* studies and by alternating the animals used for the *in vivo* studies between the first and second challenges.

#### (A) *In vitro* evaluation in rumen fluid

**Fig 1:**
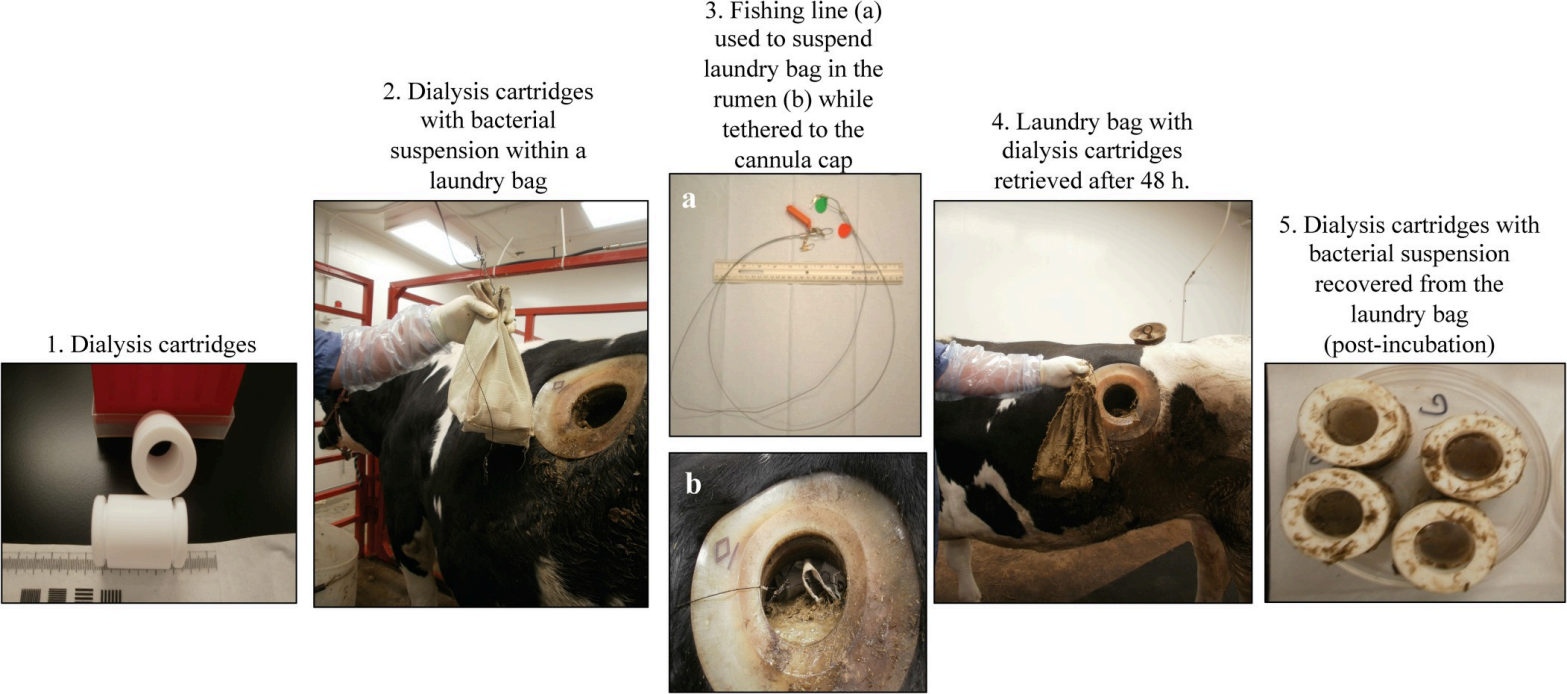
Materials and method used to introduce and retrieve bacteria from the rumen of a fistulated cow allowing reuse of the animal.

Rumen fluid collected from two different animals were mixed to account for any host-related variations in the processing of the feed and distributed in 300 ml aliquots per sterile flasks with rubber stoppers. To create anaerobic culture conditions, the flasks were transferred into the anaerobic Coy Chamber for 72 hr, charged with bacteria containing dialysis tubings or cartridges within the chamber, sealed and then removed for appropriate incubation. Dialysis tubings or cartridges fitted with membranes were used to contain the test bacteria within, allowing for easy recovery, while permitting diffusion of rumen fluid through. **(i) With dialysis tubing:** Bacteria were grown to log-phase in LB broth (OD_600_ 0.5), washed and resuspended in sterile phosphate buffered saline (PBS, pH7.4). Four ml of each bacterial suspension was added to separate sterile dialysis tubing (Spectra/Por Type F, PVDF: 80,000 kDa cut off; Serva Electrophoresis, Heidelberg, Germany). A total of 2–4 dialysis tubings per bacterial strain were then suspended in separate flasks of anaerobic rumen fluid in the Coy Chamber, prior to sealing the flasks [49]. To correlate with the feed turnover rates in the rumen [28,50], the flasks were incubated for 48 h at 39°C, anaerobically, before harvesting bacteria from the tubings for microbiological and proteomics analysis [51]. An aliquot of the aspirated bacterial suspension was used to determine colony counts. The rest of the aspirated suspension was pelleted by centrifugation at 7,000 rpm, 15 min at 4°C, washed three times with an equal volume of ice-cold sterile PBS, and processed to obtain cell lysate and pellet fractions for iTRAQ proteomics analysis [51]. **(ii) With dialysis cartridge:** Using the same experimental set up and processing conditions as described above, sterile dialysis cartridges (Ultra-test Dialyzer Chamber, Harvard Apparatus, Hollister, MA), charged with the 4 ml of the bacterial suspension in PBS and sealed at both ends with polycarbonate membranes (0.01um pore size, Harvard Apparatus), were used in place of dialysis tubings (Fig 1). A total of 2–4 dialysis cartridges per bacterial strain were used.

#### (B) *In vivo* evaluation in the rumen

Two animals were used to set up the *in vivo* studies per dietary condition. The animals were alternated between the first and second challenge to account for possible host-related influences on the bacteria besides the diet itself. To ensure retrievability from the rumen, the dialysis tubings or cartridges, charged with bacteria as described above, were placed in mesh laundry bags tethered to a 3 ft. fishing line attached to the rumen cannula cap (Fig 1). All bags with test bacteria containing dialysis tubings or cartridges were introduced into the animal. Bags were retrieved from the rumen after 48 hr. and transported to the lab on ice where the integrity of the dialysis tubings/cartridges was verified.

### Proteomics

#### (i) Protein sample preparation and labelling for Isobaric Tags for Relative and Absolute Quantification (iTRAQ) analysis

Following each *in vitro* and *in vivo* experiment, harvested bacteria of same strain type and from same growth condition, were pelleted together. The pellets were washed three times with ice-cold sterile phosphate buffered saline (PBS; pH 7.4), frozen and subsequently processed to obtain bacterial cellular and membrane proteins for proteomic analysis as described previously [51]. For iTRAQ, the protein fractions were quantified [52], and dissolved in denaturant buffer (0.1% SDS (w/v) and dissolution buffer (0.5 M triethylammonium bicarbonate, pH 8.5) in the iTRAQ 8-plex kit (AB SCIEX Inc., Foster City, CA). A total of 100 μg of protein per sample were reduced, alkylated, trypsin-digested, and resulting peptides labeled with either of the iTRAQ tags 114, 115, 116,117, 118, 119 or 121, according to the manufacturer’s instructions (AB SCIEX Inc.). Peptides from bacteria grown in MRF-*in vitro* or -*in vivo* were labeled separately based on strain-growth condition and then mixed in one tube; the LRF-*in vitro* and LRF-*in vivo* peptides were processed likewise. The mixed labeled peptides were dried and held at -80°C until ready for processing as follows. The labeled peptides were desalted with C18-solid phase extraction and dissolved in strong cation exchange (SCX) solvent A (25% (v/v) acetonitrile, 10 mM ammonium formate, and 0.1% (v/v) formic acid, pH 2.8).

#### (ii) Strong cation exchange fractionation and reverse-phase liquid chromatography with tandem mass spectrometry (LC-MS/MS)

The peptides were fractionated using an Agilent HPLC 1260 with a polysulfoethyl A column (2.1 × 100 mm, 5 μm, 300 Å; PolyLC, Columbia, MD, USA). Peptides were eluted with a linear gradient of 0–20% solvent B (25% (v/v) acetonitrile and 500 mM ammonium formate, pH 6.8) over 50 min followed by ramping up to 100% solvent B in 5 min. The absorbance at 280 nm was monitored and a total of 10 fractions per sample were collected. The fractions were lyophilized and resuspended in LC solvent A (0.1% formic acid in 97% water (v/v), 3% acetonitrile (v/v)). A hybrid quadrupole Orbitrap (Q Exactive Plus) MS system (Thermo Fisher Scientific, Bremen, Germany) was used with high energy collision dissociation (HCD) in each MS and MS/MS cycle. The MS system was interfaced with an automated Easy-nLC 1000 system (Thermo Fisher Scientific). Each sample fraction was loaded onto an Acclaim Pepmap 100 pre-column (20 mm × 75 μm; 3 μm-C18) and separated on a PepMap RSLC analytical column (250 mm × 75 μm; 2 μm-C18) at a flow rate at 350 nl/min during a linear gradient from solvent A (0.1% formic acid (v/v)) to 30% solvent B (0.1% formic acid (v/v) and 80.0% acetonitrile (v/v)) for 95 min, to 98% solvent B for 15 min, and hold 98% solvent B for additional 30 min. Full MS scans were acquired in the Orbitrap mass analyzer over m/z 400–2000 range with resolution 70,000 at 200 m/z. The top ten most intense peaks with charge state ≥ 2 were isolated (with 2 m/z isolation window) and fragmented in the high energy collision cell using a normalized collision energy of 28% selected. The maximum ion injection time for the survey scan and the MS/MS scans were 250 ms, and the ion target values were set to 3e6 and 1e6, respectively. The selected sequenced ions were dynamically excluded for 60 sec.

### Intensity Based Absolute Quantification (iBAQ) analysis of the LC-MS/MS data

Label-free iBAQ values for each protein within each rumen fluid were determined using MaxQuant to analyze the raw LC-MS/MS data [53]. For protein identification, a custom *E*. *coli* database was generated by combining protein sequences from 162 O157 genomes available from NCBI and is available in the supplementary file: NCBI_Ecoli_used_for_reference.txt. Redundant proteins were collapsed with CD-HIT [54]. Additionally, we included the Uniprot *E*. *coli* panproteome UP000000625 [55] to ensure the most comprehensive reference possible. MaxQuant was run using the default parameters, with the selection of calculating label free quantification iBAQ values; detailed information about the parameters used is available in supplementary file: MQ_O157_parameters.txt. iBAQ values were normalized, using R scripts, by dividing them by the sum of iBAQs in the sample to obtain relative iBAQs (riBAQs) [56]. These riBAQ values were used to compare relative protein abundances between the two different rumen fluids. We calculated log2 fold change (L2FC) values between MRF and LRF using riBAQ values (Log2(riBAQlrf/riBAQmrf)) and considered any protein with an absolute L2FC value of >1 to be enriched in the respective rumen fluid.

### Isobaric Tags for Relative and Absolute Quantitation (iTRAQ) analysis of the LC-MS/MS data

#### (i) Database searching

Tandem mass spectra were extracted by Proteome Discoverer (Thermo-Fisher) version 2.2.0.388. Charge state deconvolution and deisotoping were not performed. All MS/MS samples analyzed were processed by a thorough database searching considering biological modification and amino acid substitution against a non-redundant Uniprot *E*. *coli* strain K12 database (4,448 entries), *E*. *coli* O157:H7 strain EDL933, UP000028484 (5,270 entries) and *E_coli*_O157_20160822 database (75243 entries) with decoy option using MASCOT 2.4 (Matrix Science Inc., Boston, MA, USA) assuming the digestion enzyme trypsin. Mascot was searched with a fragment ion mass tolerance of 0.6–0.02 Da and a parental ion tolerance of 20–10 ppm. Methylthio of cysteine and iTRAQ8plex of lysine and the n-terminus were specified in Mascot as fixed modifications. Gln->pyro-Glu of the n-terminus, deamidated of asparagine and glutamine, methyl of lysine, oxidation of methionine, phospho of serine, threonine and tyrosine, nmethylmaleimide of cysteine and iTRAQ8plex of tyrosine were specified in Mascot as variable modifications.

#### (ii) Criteria for protein identification

Scaffold (version Scaffold_4.8.7, Proteome Software Inc., Portland, OR) was used to validate MS/MS based peptide and protein identifications. Peptide identifications were accepted if they could be established at greater than 95.0% probability by the Peptide Prophet algorithm [57] with Scaffold delta-mass correction. Protein identifications were accepted if they could be established at greater than 95.0% probability and contained at least 2 identified peptides. Protein probabilities were assigned by the Protein Prophet algorithm [58]. Proteins that contained similar peptides and could not be differentiated based on MS/MS analysis alone were grouped to satisfy the principles of parsimony. Proteins sharing significant peptide evidence were grouped into clusters.

#### (iii) Data analysis of peptides/proteins comprising the O157 proteome expressed in rumen fluid from animals fed the maintenance diet

Scaffold Q+ (version Scaffold_4.8.7, Proteome Software Inc., Portland, OR) was used to quantitate Label Based Quantitation (iTRAQ, TMT, SILAC, etc.) peptide and protein identifications. Peptide identifications were accepted if they could be established at greater than 95.0% probability by the Peptide Prophet algorithm [57] with Scaffold delta-mass correction. Protein identifications were accepted if they could be established at greater than 95.0% probability and contained at least 2 identified peptides. Protein probabilities were assigned by the Protein Prophet algorithm [58]. Proteins that contained similar peptides and could not be differentiated based on MS/MS analysis alone were grouped to satisfy the principles of parsimony. Proteins sharing significant peptide evidence were grouped into clusters. The false discovery rate (FDR) at the peptide level was set with the integrated PSPEP tool in the Protein Pilot Software to be at 1.0%. Channels were corrected by the matrix [0.000,0.000,0.929,0.0689,0.00220]; [0.000,0.00940,0.930,0.0590,0.00160]; [0.000,0.0188,0.931,0.0490,0.001000]; [0.000,0.0282,0.932,0.0390,0.000700]; [0.000600,0.0377,0.933,0.0288,0.000]; [0.000900,0.0471,0.933,0.0188,0.000]; [0.00140,0.0566,0.933,0.00870,0.000]; [0.000,0.000,0.000,0.000,0.000]; [0.00270,0.0744,0.921,0.00180,0.000] in all samples according to the algorithm described in i-Tracker [59]. Normalization was performed iteratively (across samples and spectra) on intensities, as described in Statistical Analysis of Relative Labeled Mass Spectrometry Data from Complex Samples Using ANOVA [60]. Medians were used for averaging. Spectra data were log-transformed, pruned of those matched to multiple proteins and those missing a reference value, and weighted by an adaptive intensity weighting algorithm. Of 9136 spectra in the experiment at the given thresholds, 5895 (65%) were included in quantitation.

#### (iv) Data analysis of peptides/proteins comprising the O157 proteome expressed in rumen fluid from animals fed the lactation diet

Scaffold Q+ (version Scaffold_4.8.7, Proteome Software Inc., Portland, OR) was used to quantitate Label Based Quantitation (iTRAQ, TMT, SILAC, etc.) peptide and protein identifications. Peptide identifications were accepted if they could be established at greater than 95.0% probability by the Peptide Prophet algorithm [57] with Scaffold delta-mass correction. Protein identifications were accepted if they could be established at greater than 95.0% probability and contained at least 2 identified peptides. Protein probabilities were assigned by the Protein Prophet algorithm [58]. Proteins that contained similar peptides and could not be differentiated based on MS/MS analysis alone were grouped to satisfy the principles of parsimony. Proteins sharing significant peptide evidence were grouped into clusters. FDR at the peptide level was set with the integrated PSPEP tool in the Protein Pilot Software to be at 1.0%. Normalization was performed iteratively (across samples and spectra) on intensities, as described in Statistical Analysis of Relative Labeled Mass Spectrometry Data from Complex Samples Using ANOVA [60]. Medians were used for averaging. Spectra data were log-transformed, pruned of those matched to multiple proteins and those missing a reference value, and weighted by an adaptive intensity weighting algorithm. Of 4924 spectra in the experiment at the given thresholds, 4739 (96%) were included in quantitation.

#### (v) iTRAQ Quantitation with (iTRAQ analysis #1) or without (iTRAQ analysis #2) reference comparison

For quantification, only MS/MS spectra that were unique to a particular protein and where the sum of the signal-to-noise ratios for all the peak pairs at > 9 were used. The ratios with *p*-values less than 0.05 present in at least two replicates were considered significant. Only the significant ratios from the replicates were used to calculate the average ratio for the protein. For a protein to be determined as differentially expressed, it must have been identified and quantified with at least two unique peptides. The iTRAQ quantitation was done either in comparison to a reference (iTRAQ analysis #1) or in a reference-free manner (iTRAQ analysis #2).

For iTRAQ analysis #1, individual proteomes expressed by the strains, *in vitro* or *in vivo*, were compared separately against the proteome expressed by the O157 strain 86–24 under *in vitro* conditions (reference proteome) for each type of rumen fluid used in this study. Any O157 protein with L2FC, set in Scaffold to a minimum +/- 1 FC, equaling a 2-fold change in comparison to the reference was determined to be differentially expressed in the *in vitro* or *in vivo* growth conditions, in either MRF or LRF. The total L2FC value determined for each protein in the 10 fractions per strain sample was used to determine differential expression.

For iTRAQ analysis #2, reporter ion intensities across the strains were compared, for the *in vitro* or *in vivo* growth conditions, in each type of rumen fluid. The reporter intensities were averaged for all three strains in each growth condition, then the formula Log2(AVG_vitro / AVG_vivo) used for the final L2FC calculation. Differentially expressed proteins, between *in vitro* and *in vivo* conditions, in MRF and LRF were determined by applying Mann-Whitney Test with unadjusted significance level *p* < 0.05 corrected by Benjamini-Hochberg.

### Statistics and Bioinformatics

The 2-way ANOVA test was used to compare VFA concentrations and a value of *p*<0.05 was considered significant. Several different methods were used to gain biological insights into the proteins enriched under various conditions. First, the predicted cellular location of all proteins was determined using PSORTb v3.0.2 [51,61]. Next, Gene Ontology (GO) terms to all proteins were annotated in the reference databases with InterProScan5 [62]. For sets of differentially expressed proteins enriched GO-terms were calculated with the R package TopGO [63–66]. The R package vegan [67] was used to perform multivariate statistical tests on the composition and dispersion of protein expression patterns between various conditions. The complete R script used to perform statistical test and figure generation is available at https://github.com/USDA-FSEPRU/O157_rumen_proteomics_1/.

## Results

### Rumen fluid pH and VFA concentrations varied with diet

**Fig 2:**
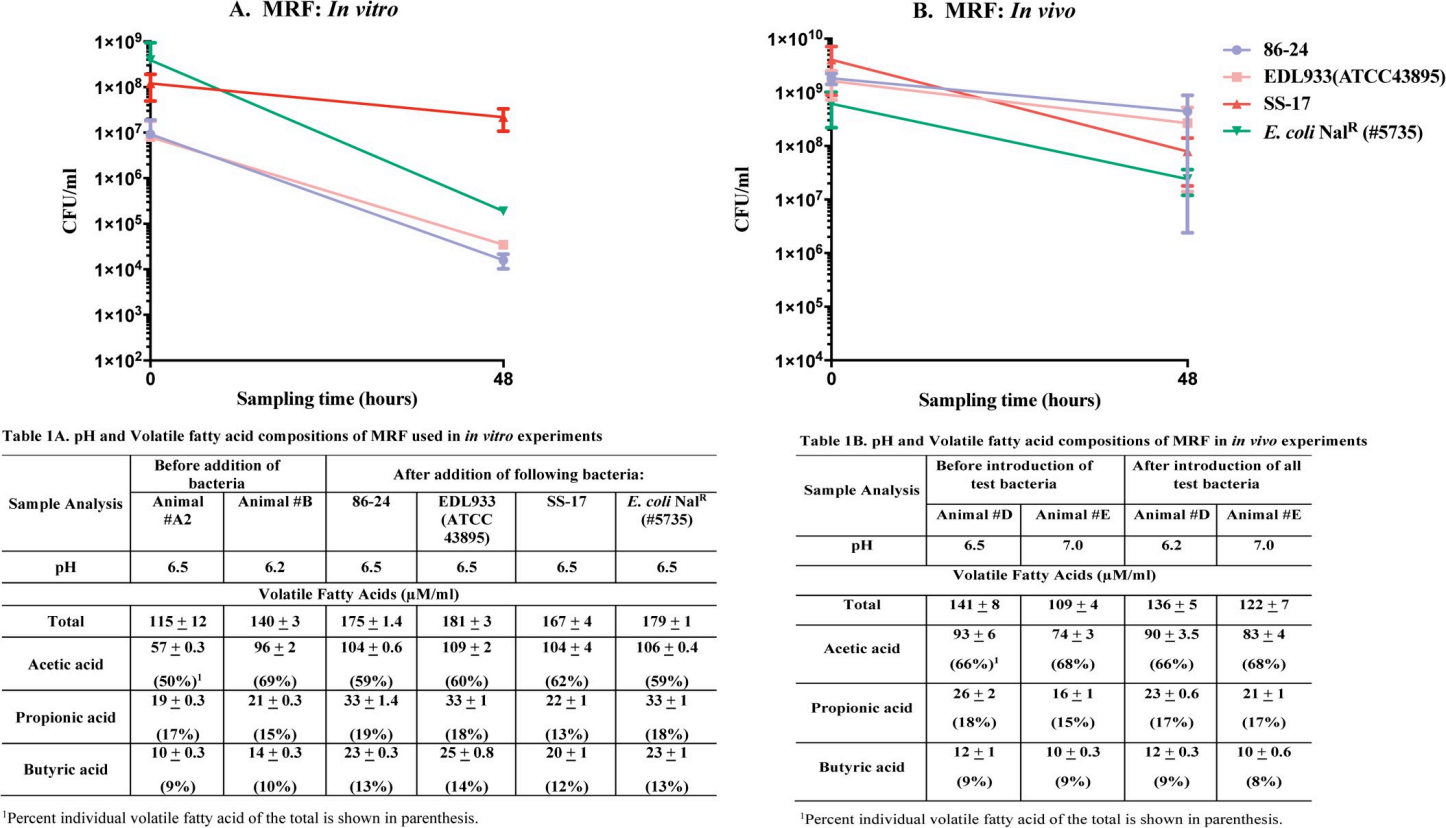
Graphs shown represent survival characteristics of three O157 strains in comparison to *E*. *coli* Nal^R^ (#5735), *in vitro* (A) and *in vivo* (B), in MRF. Bacterial survival characteristics depicted are following anaerobic incubation for 48 h, *in vitro* in flasks with MRF or *in vivo* in the rumen of animals fed the maintenance diet. Viable counts in colony forming units [cfu]/ml, with the standard error of means, are shown in both graphs. Key for each bacterial strain tested is also shown. Inserted tables show pH and VFA composition of MRF used in the *in vitro* (1A) and *in vivo* (1B) experiments.

**Fig 3:**
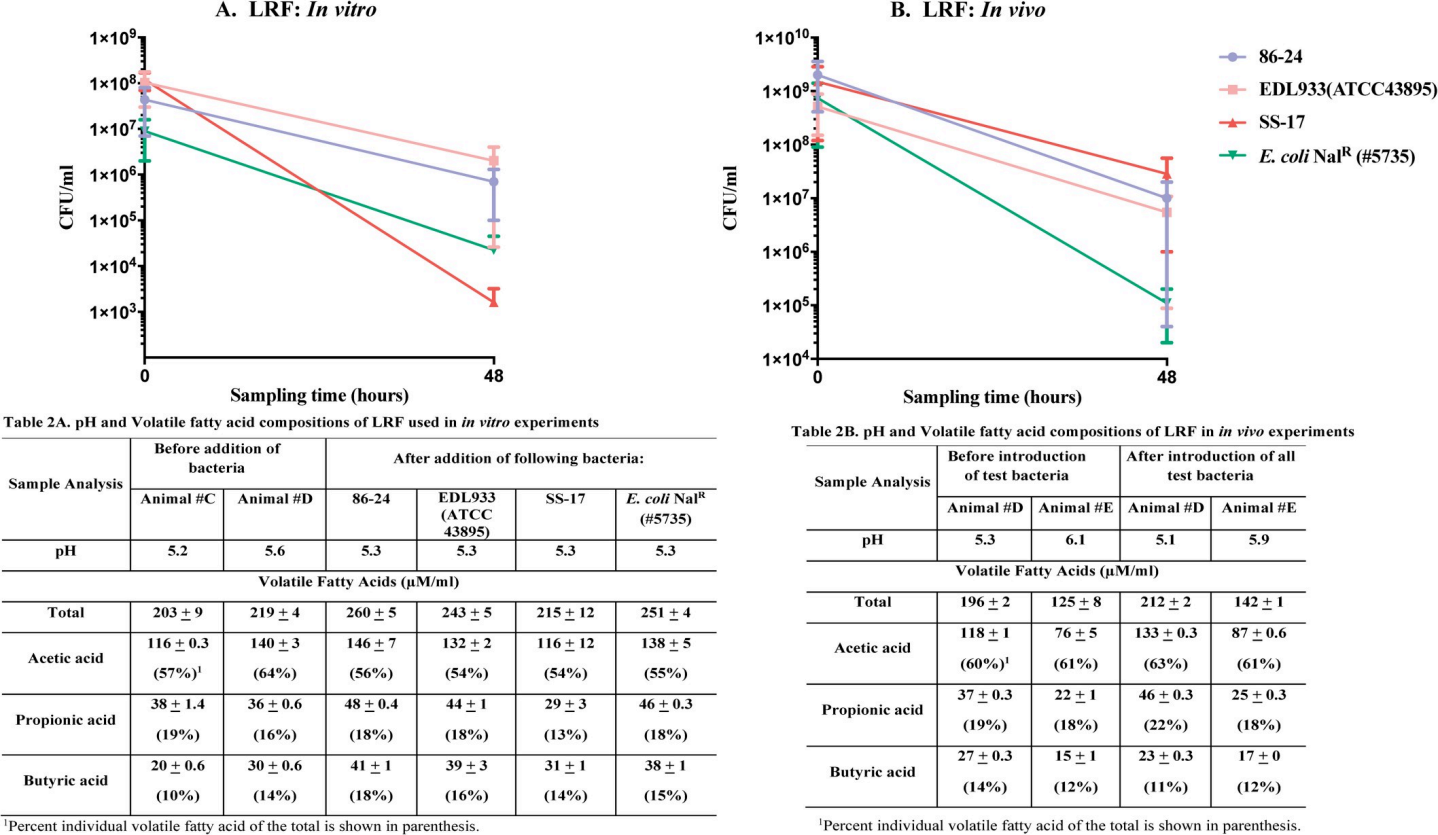
Graphs shown represent survival characteristics of three O157 strains in comparison to *E*. *coli* Nal^R^ (#5735), *in vitro* (A) and *in vivo* (B), in LRF. Bacterial survival characteristics depicted are following anaerobic incubation for 48 h, *in vitro* in flasks with LRF or *in vivo* in the rumen of animals fed the lactation diet. Viable counts in colony forming units [cfu]/ml, with the standard error of means, are shown in both graphs. Inserted tables show pH and VFA composition of LRF used in the *in vitro* (2A) and *in vivo* (2B) experiments.

The two diets had differing influences on the ruminal pH and volatile fatty acid (VFA) profiles (Table 1A and 1B in Fig 2 and Table 2A and 2B in Fig 3). The concentrations of three VFAs, acetate, propionate and butyrate that vary the most with changes in diet and influence bacterial and host growth, were closely analyzed in the context of the total VFA [28,48].

**(i) In MRF.** In the *in vitro* experiments, as shown in Table 1A in Fig 2, the ruminal pH ranged from 6.2 to 6.5 irrespective of the presence of the test bacteria. The total VFA concentrations ranged between 115–181 μM/ml, among animals fed the maintenance diet, with slightly increased VFA post-exposure to test bacteria. In the *in vivo* experiments, the ruminal pH ranged from 6.2 to 7.0 and the total VFA between 109–141 μM/ml (Table 1B in Fig 2).

**(ii) In LRF.** In the *in vitro* experiments, the ruminal pH ranged from 5.2–5.6, without any influence from the test bacteria (Table 2A in Fig 3). The total VFA concentrations ranged from 203–260 μM/ml; higher values were observed in post-exposure to test bacteria (Table 2A in Fig 3). Likewise, *in vivo*, the ruminal pH ranged from 5.1–6.1 and total VFA concentrations were between 125–212 μM/ml (Table 2B in Fig 3). Percent differences in butyrate concentration were more pronounced *in vivo* in LRF (Table 1B in Fig 2 and Table 2B in Fig 3; 8–9% in MRF versus 11–14% in LRF) as expected with a high energy diet [39,40]. In addition, total VFA concentrations were significantly different (*p<*0.0001) between LRF and MRF and may have contributed to the decrease in pH in this rumen fluid compared to MRF.

### Dialysis cartridges enabled the development of a novel, non-terminal method for *in vivo* evaluation of bacteria in the bovine rumen

Dialysis tubings were compared against dialysis cartridges with membranes for the ability to retain test bacterial cultures within while maintaining structural integrity to prevent inadvertent spillage into the rumen fluid in the flask or the cattle rumen in the pilot study. The animals were fed the maintenance diet for this entire experiment. Saline suspensions (4 ml/tubing or cartridge) of log-phase *E*. *coli* Nal^R^ (#5735), a non-pathogenic bacteria, were used to charge both the tubings and cartridges as described above under *Materials and Methods*.

#### (i) *In vitro* pilot study

The rumen fluid obtained from animal #A was at pH 6.63 with 54% acetate, 13% propionate, and 8% butyrate. Comparable results, both with bacterial counts and volume of inoculum recovered in 48 h, were observed while using dialysis tubings and cartridges to introduce *E*. *coli* Nal^R^ (#5735) into the rumen fluid in flasks (S1 Table). As shown in supplementary S1 Table, average of counts obtained from two tubings or cartridges indicated minimal change in viable counts post-anaerobic incubation for 48 h. No leakage of *E*. *coli* Nal^R^ (#5735), from the intact tubings and cartridges charged with the inoculum, into the surrounding rumen fluid was observed following culture of the same.

#### (ii) *In vivo* pilot study

The rumen fluid in animal #A was at pH 7.06 with 68% acetate, 15% propionate and 9% butyrate at the time of this study. The culture results of this study are shown in the S1 Table and represent average of counts obtained from two tubings or cartridges. Using the dialysis tubing an average of 2-log reduction in the *E*. *coli* Nal^R^ (#5735) viable count was observed compared to a 1-log reduction with the dialysis cartridges after being *in vivo* in the bovine rumen for 48h (S1 Table). Of the 2 dialysis tubings introduced into the rumen, only one remained intact and the initial volume of 4 ml was reduced to 200μl; most of the inoculated bacteria was adherent on the tubing material itself. The base of one dialysis tubing was shred in the rumen, and we recovered *E*. *coli* Nal^R^ (#5735) only from the bovine feces, post-enrichment in LB-Nal broth, at the 48h sampling. In contrast to the tubings, both the dialysis cartridges remained intact, and 3 ml of the 4 ml initial volume was recovered from each of the cartridges. *E*. *coli* Nal^R^ (#5735) was found adhering to the walls of the cartridges as with the dialysis tubing. All adherent bacteria were scraped off the tubing or cartridge walls, using sterile inoculum loops, into the suspension within before aspirating the same. Overall, the dialysis cartridges performed better than the PVDF tubings in the *in vivo* experiment as the cartridges were better at resisting the shear forces of ruminal contractions. No leakage of *E*. *coli* Nal^R^ (#5735), from the cartridges charged with the inoculum, into the rumen of the animal was observed based on the intact assembly of the cartridge and negative rumen fluid- and fecal- cultures over two weeks post-experiment.

### O157 strains demonstrated variable survival in MRF and LRF both *in vitro* and *in vivo*

Based on the results of the pilot study, dialysis cartridges were used for all the experiments testing bacterial strains simultaneously. For *in vitro* assays, rumen fluid collected from two animals was mixed prior to distribution into flasks to account for possible host-based variations. In case of *in vivo* assays, each of the duplicate experiments was conducted in separate animals for similar reasons. Strain dependent differences in O157 survival were observed in the rumen fluid of cattle fed either diet, in both the *in vitro* and *in vivo* assays, with O157 strains 86–24 and EDL933 demonstrating similar recovery patterns. In comparison, O157 strain SS-17 and the non-STEC *E*. *coli* Nal^R^ (#5735) had variable survival in either assay condition.

#### (i) *In vitro* survival characteristics in rumen fluid

Following anaerobic incubation in MRF, in flasks at 39°C for 48 h, O157 strains 86–24 and EDL933 demonstrated 2-log reduction and O157 strain SS-17 a 1-log reduction in viable counts compared to the 3-log reduction of *E*. *coli* Nal^R^ (#5735) viable counts (Fig 2; S2 Table). Rumen fluid from one animal was used for the pilot-*in vitro* experiment while rumen fluid from two different animals was mixed for the test-*in vitro* experiment. This may have presented more comprehensive growth inhibitory factors resulting in the discrepancy in the *E*. *coli* Nal^R^ (#5735) viable counts recovered at 48h between the pilot and test experiments (S1 and S2 Tables). In LRF, after similar incubation, O157 strains 86–24 and EDL933 had a 2-log reduction and *E*. *coli* Nal^R^ (#5735) a 1-log reduction in viable counts while O157 strain SS-17 had a 5-log decrease in viable counts (Fig 3; S3 Table). Based on these results, it appeared that O157 strain SS-17 was more sensitive to the low pH, higher VFA conditions, *in vitro*, in LRF than the other O157 strains. None of the O157 strains tested were recovered from the rumen fluid in the flasks, prior to and post exposure to the bacteria in dialysis cartridges.

#### (ii) *In vivo* survival characteristics in the rumen

Following 48 h incubation within the rumen of fistulated cows fed the maintenance diet, the viable counts of O157 strains 86–24 and EDL933, and *E*. *coli* Nal^R^ (#5735) reduced by 1-log compared to the 2-log reduction in counts of O157 strain SS-17 (Fig 2; S2 Table). *E*. *coli* Nal^R^ (#5735) counts recovered at 48h were consistent between the pilot- and test- *in vivo* experiments verifying the consistency of an *in vivo* system, and a more dietary than host- related influence on bacterial gene expression since different animals were used for these experiments (S1 and S2 Tables). Interestingly, *in vivo* in the rumen of animals fed the lactation diet, all O157 strains shared growth patterns that grouped them apart from *E*. *coli* Nal^R^ (#5735); viable counts decreased by 2-logs for all O157 strains and by 3-logs for *E*. *coli* Nal^R^ (#5735) (Fig 3; S3 Table). These results differed from the *in vitro* assays which further verifies the importance of conducting *in vivo* experiments as some cues present in a live host can be missed in *in vitro* systems. None of the O157 strains tested were recovered from the rumen fluid and fecal samples of the animals used in the study prior to and post exposure to the bacteria in dialysis cartridges.

### Comprehensive analysis of proteomics data allowed identification of O157 proteins uniquely or differentially expressed under varying growth conditions in MRF and LRF

All analysis was done in the context of the diets influencing growth of O157 strains in the rumen fluid *in vitro*, or *in vivo* in the bovine rumen.

#### (i) iBAQ analysis identified O157 proteins uniquely expressed, in MRF and LRF

**Fig 4:**
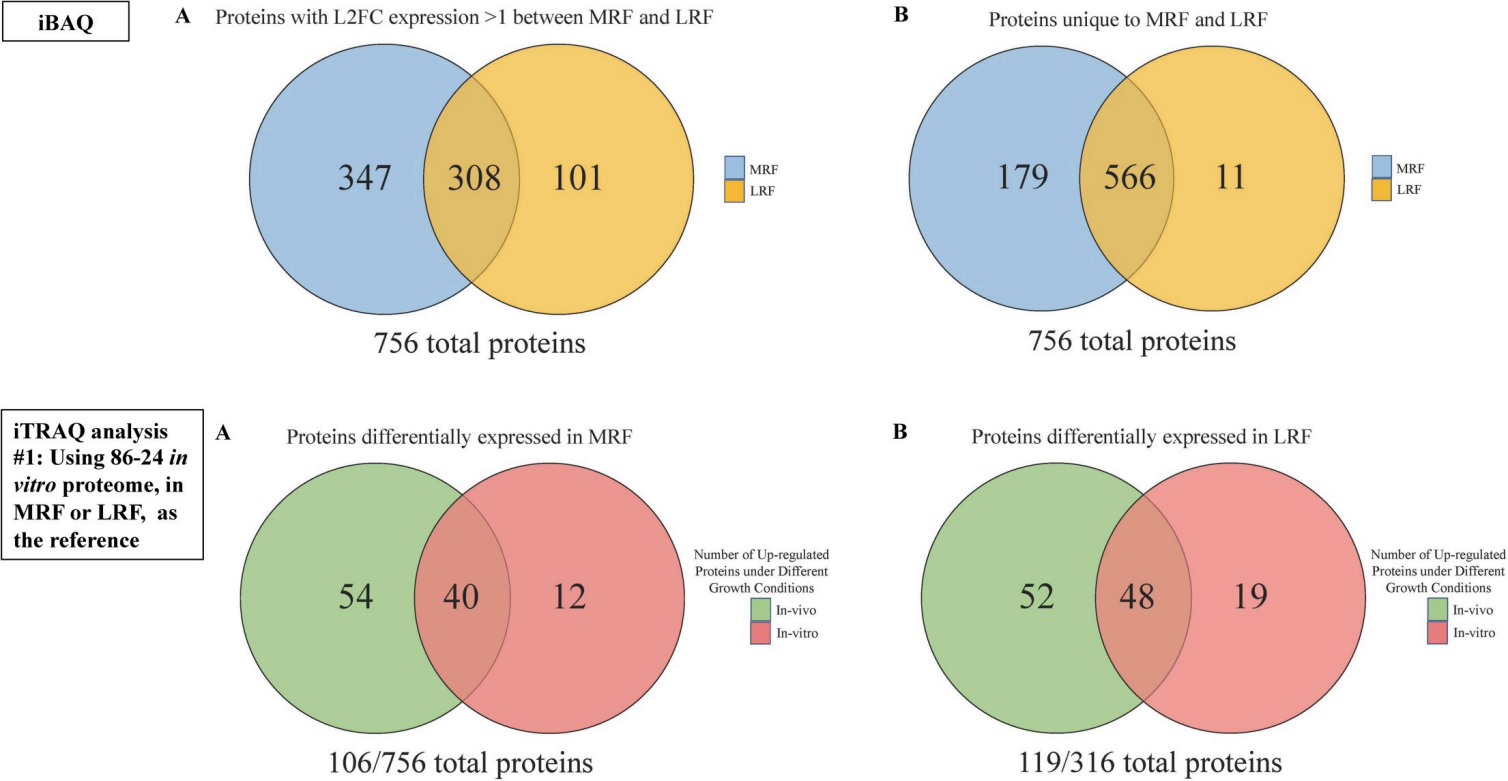
Analysis of O157 proteome as expressed in MRF and LRF, under different diet and growth conditions, using iBAQ and reference-based iTRAQ analysis #1. **iBAQ****: A.** Venn diagram showing the number of O157 proteins differentially expressed (L2FC >1) between MRF and LRF. **B.** Venn diagram showing the number of O157 proteins uniquely expressed in MRF and LRF. **iTRAQ analysis #1 (reference-based):** **A.** Venn diagram showing number of differentially expressed proteins in the *in vitro* and *in vivo* growth conditions, relative to the 86–24 *in-vitro* proteome, in MRF. **B.** Venn diagram showing number of differentially expressed proteins in the *in vitro* and *in vivo* growth conditions, relative to the 86–24 *in-vitro* proteome, in LRF.

iBAQ analysis was used to determine overall differences in O157 proteins expressed, differentially or uniquely, in MRF versus LRF without focusing on individual strains or the *in vitro*/*in vivo* growth conditions. Since the iTRAQ experiments were designed to give the highest resolution of differences between growth conditions (*in vitro* versus *in vivo*) within each rumen fluid (all iTRAQ labeled MRF samples were mixed and analyzed in one MS run, and all iTRAQ labeled LRF samples in another), the iTRAQ reporter intensity could not be used effectively to compare protein expression between the rumen fluids. However, because all samples pooled in each MS run belonged to the same rumen fluid type, iBAQ values of the same, which excludes labels and merges all data for a particular rumen fluid type, could be used to estimate the differences in protein expression between MRF and LRF. These iBAQ values represented pooled protein expression for all three O157 strains in both the *in-vitro* and *in-vivo* culture conditions for each rumen fluid. A much lower total iBAQ value was detected for the O157 proteome in LRF, which may be reflective of the decrease in viable counts of the strains grown under these conditions (S3A Fig). All the same, to avoid any false interpretations due to differences in intensities, iBAQ values were converted to riBAQ values to look at relative protein abundance that is, proportion of each protein within the total protein pool, for each rumen fluid type [68] (S3B Fig; S4A Table). A total of 756 O157 proteins were identified by riBAQ of which 566 proteins were detected in both rumen fluids, 179 were unique to MRF and 11 unique to LRF (Fig 4; S4B Table). The L2FC values were calculated between the two rumen fluids using riBAQ values and any protein with an absolute L2FC value of 1 or greater was considered to be enriched in the respective rumen fluid (Fig 4). Using these criteria 347 O157 proteins were found to be enriched in MRF (S4C Table) and 101 enriched in LRF (S4D Table). These differentially represented O157 proteins were used in a GO term enrichment analysis to identify GO terms that were significantly enriched in each rumen fluid (S5A and S5B Table).

#### (ii) Reference-based iTRAQ analysis #1 identified O157 proteins differentially expressed under *in vitro* and *in vivo* growth conditions relative to the 86–24 *in vitro* proteome, in MRF or LRF

Post-application of the filtration (protein probability 95%, minimum number of peptides 2, peptide probability 95%) criteria as described in the *Materials and Methods* section above, a total of 756 and 316 O157 proteins were identified as being expressed, cumulatively, by the three O157 strains in MRF and LRF, respectively. Application of the quantitation criteria (minimum +/- 1 FC equaling a 2-fold change, in comparison to the previously reported [28] reference O157 strain 86–24 proteome expressed *in vitro* in MRF or LRF) to the total number of O157 proteins enabled the identification of proteins differentially expressed in the *in vitro* or *in vivo* growth conditions, with either MRF or LRF. After excluding duplicate hits and proteins with a value of absolute zero (no change) a cumulative list of differentially expressed proteins across the O157 strains was generated. Comparatively, slightly more proteins were differentially expressed in RF from animals fed the lactation diet (119 out of a total of 316 proteins; 38%) than in RF from animals fed the maintenance diet (106 out of a total of 756 proteins; 14%) (S6A and S6B Table). These differences in protein expression were not always O157 strain-specific as majority of the proteins were either up- or down-regulated across all three O157 strains tested with a few exceptions.

Proteins with a FC ≥ +1, in either of the three O157 proteomes relative to the 86–24 *in vitro* proteome, were designated as up-regulated for the specific growth condition. Taking these up-regulated proteins into account, of the 106 O157 proteins differentially expressed in MRF, 12 were found to be highly expressed in the *in vitro* condition, 54 were highly expressed in the *in vivo* condition while the remaining 40 were either down-regulated or equally regulated under both growth condition (Fig 4, Table 1A and 1B). Likewise, of the 119 O157 proteins differentially expressed in LRF, 19 were highly expressed *in vitro*, 52 were highly expressed *in vivo* and 48 were down-regulated or equally regulated under both growth condition (Fig 4, Table 1C and 1D).

**Table 1:** O157 proteins up-regulated, under the *in vitro* and *in vivo* growth conditions, in MRF and LRF, as determined by reference-based iTRAQ analysis #1. (Reference:86–24 *in vitro* proteome).

**A. MRF *In vitro*:**			
General function/ Protein name^1^	Specific function	Bacterial-cell localization	Accession number
Lipid and carbohydrate metabolism			
MalK	Maltose ABC transporter ATP binding subunit/ starch and sucrose metabolism; transport	Inner membrane	A0A0F6CBZ6_ECOLX
MalP	Maltodextrin phosphorylase/ trehalose, starch, and sucrose metabolism	Cytoplasm	J7QI49_ECOLX
Protein and nucleotide metabolism			
Pnp	Polynucleotide phosphorylase/ RNA catabolism and tRNA processing	Cytoplasm	A0A061K888_ECOLX
Energy metabolism			
DkgA	Methylglyoxal reductase/ ketogluconate metabolism	Cytoplasm	A0A0F6C8Y9_ECOLX
PckA	Phosphoenolpyruvate carboxykinase (ATP)/ gluconeogenesis	Cytoplasm	A0A037YEU7_ECOLX
Regulatory			
Hfq	RNA-binding protein/ transcription and translation regulator	Cytoplasm	A0A0F6CCC8_ECOLX
PspA	Phage shock protein/ transcription regulator	Inner	A0A0F6C3Q2_ECOLX
		Membrane	
Transcription and translation			
**FkpA**	Peptidyl-prolyl cis-trans isomerase/ protein folding	Periplasm	A0A024L0A6_ECOLX
**HupA**	DNA-binding protein HU-alpha/ DNA transcription	Cytoplasm	A0A0F6CBV8_ECOLX
**RplU**	50S ribosomal subunit protein L21/ protein translation	Cytoplasm	A0A0F6C9F6_ECOLX
**RpsD**	30S ribosomal subunit protein S4/ protein translation	Cytoplasm	A0A077K4W3_ECOLX
**RpsG**	30S ribosomal subunit protein S7/ protein translation	Cytoplasm	A0A0F6C9T4_ECOLX
**B. MRF *In vivo*:**			
General function/ Protein name^1^	Specific function	Bacterial-cell localization	Accession number
Lipid and carbohydrate metabolism			
AccC	Biotin carboxylase/ fatty acid biosynthesis	Cytoplasm	A0A066REV0_ECOLX
RbiA	Ribose-5-phosphate isomerase A	Cytoplasm	A0A0F6C8N8_ECOLX
Protein metabolism			
DegQ	Periplasmic serine endoprotease/ proteolysis	Periplasm	A0A0F3UJ52_ECOLX
**LeuB**	3-isopropylmalate dehydrogenase/ leucine biosynthesis	Cytoplasm	C3TQW2_ECOLX
OmpT	Outer membrane protease VII (outer membrane protein 3b)/ proteolysis	Outer membrane	A0A0C2BHT4_ECOLX
PepA	Aminopeptidase A/I/ protein metabolism; proteolysis	Cytoplasm	A0A0F6CCL5_ECOLX
Prc	Tail-specific protease/ proteolysis; signal transduction; antibiotic response	Plasma membrane	A0A0D0NMS5_ECOLX
SpeB	Agmatinase/ polyamine and putrescine biosynthesis; arginine metabolism	Cytoplasm	A0A0F6C8R3_ECOLX
Energy metabolism			
**AceF**	Pyruvate dehydrogenase, E2 subunit/ energy metabolism	Cytoplasm	A0A0F6BYW1_ECOLX
AcnB	Aconitase B/ tricarboxylic acid cycle; translation regulator	Cytoplasm	A0A0H3PMJ2_ECO5C
Apt	Adenine phosphoribosyltransferase/ nucleoside metabolism	Cytoplasm	A0A0F6C000_ECOLX
Can	Carbonic anhydrase 2/ carbon utilization	Cytoplasm	A0A0F6BYX2_ECOLX
CydA	Cytochrome bd-I ubiquinol oxidase subunit I/ aerobic electron transport chain; oxidative phosphorylation	Inner membrane	A0A0D0LX27_ECOLX
Dfp	Fused 4’-phosphopantothenoylcysteine decarboxylase and phosphopantothenoylcysteine synthetase/ coenzyme A biosynthesis; metabolism	Cytoplasm	C3SMD2_ECOLX
Eda	KHG/KDPG aldolase/ Entner-Doudoroff pathway; metabolism	Cytoplasm	A0A0F6C5E4_ECOLX
FldA	Flavodoxin 1/ electron transport chain; nitrogen metabolism	Cytoplasm	A0A0F6C0I6_ECOLX
GltA	Citrate synthase/ metabolism; electron transport chain	Cytoplasm	E2QI94_ECOLX
Gpt	Xanthine-guanine phosphoribosyltransferase/ nucleoside metabolism; GMP and IMP salvage	Cytoplasm/Inner membrane	A0A0F6BZA5_ECOLX
PurE	N(5)-carboxyaminoimidazole ribonucleotide mutase/ purine and inosine biosynthesis	Cytoplasm	A0A0F6C058_ECOLX
PyrH	UMP kinase/ nucleotide interconversion; metabolism	Cytoplasm	A0A0F6BZ15_ECOLX
SucC	Succinyl-CoA synthetase subunit beta/ tricarboxylic acid cycle	Cytoplasm	A0A0F6C0M4_ECOLX
SucD	Succinyl-CoA synthetase subunit alpha/ tricarboxylic acid cycle	Cytoplasm	A0A0F6C0M5_ECOLX
YhcB	Cytochrome d ubiquinol oxidase subunit III/ oxidative phosphorylation; energy	Inner membrane	K3LCL2_ECOLX
UbiD	3-octaprenyl-4-hydroxybenzoate decarboxylase/ ubiquinone biosynthesis	Cytoplasm/Inner membrane	A0A0F6CBF5_ECOLX
Regulatory			
CreA	Catabolite regulation protein A/ global regulator; carbon catabolism	Cytoplasm	A0A0F6CCX8_ECOLX
Fur	DNA-binding transcriptional dual regulator/ transcription regulator	Cytoplasm	K4Y7I2_ECOLX
Transcription and translation			
ArgS	Arginine-tRNA ligase/ protein translation; arginyl-tRNA aminoacylation	Cytoplasm	E2QN80_ECOLX
**CspC**	Stress protein/ negative DNA transcription regulator; gene expression	Cytoplasm	A0A0F6C5B6_ECOLX
**GlnS**	Glutamine-tRNA ligase/ protein translation; glutaminyl-tRNA aminoacylation	Cytoplasm	A0A0F6C0I2_ECOLX
RplE	50S ribosomal subunit protein L5/ protein translation	Cytoplasm	A0A077K645_ECOLX
RplI	50S ribosomal subunit protein L9/ protein translation	Cytoplasm	A0A0F6CCF9_ECOLX
RplK	50S ribosomal subunit protein L11/ protein translation	Cytoplasm	A0A0F6CBU0_ECOLX
RpmC	50S ribosomal subunit protein L29/ protein translation	Cytoplasm	A0A077K4U3_ECOLX
RpoA	RNA polymerase subunit alpha/ DNA transcription	Cytoplasm	A0A0F6C9Q1_ECOLX
**RpsF**	30S ribosomal subunit protein S6/ protein translation	Cytoplasm	A0A0F6CCF6_ECOLX
**TrpS**	Tryptophan-tRNA ligase/ protein translation	Cytoplasm	D3QTK1_ECOCB
ValS	Valine-tRNA ligase/ protein translation; valyl-tRNA aminoacylation	Cytoplasm	A0A0F3U2Y9_ECOLX
Transport			
**DppA**	Dipeptide ABC transporter periplasmic binding protein/ inner membrane transport; chemotaxis	Periplasm	A0A0D8WAA9_ECOLX
FeoA	Ferrous iron transport protein A/ iron transport	Cytoplasm	A0A0F3U828_ECOLX
**FhuA**	Ferrichrome outer membrane transporter- phage receptor/ ion and siderophore transport	Outer membrane	K3KZG8_ECOLX
GtdA	Gentisate 1, 2-dioxygenase/ ion binding and transport	Cytoplasm	A0A0B0VQI2_ECOLX
MdtE	Multidrug efflux pump membrane fusion protein/ bile acid and salt transport; antibiotic response	Inner	K3JKS3_ECOLX
		Membrane	
**ModA**	Molybdate ABC transporter periplasmic binding protein/ molybdate transport	Periplasm	A0A0F3UDV0_ECOLX
**PotD**	Spermidine- putresceine ABC transporter/ periplasmic binding protein/ transport	Periplasm	A0A023KW13_ECOLX
Cell membrane			
LpoA	Outer membrane lipoprotein-activator of MrcA activity/ peptidoglycan synthesis	Outer membrane	A0A066QX06_ECOLX
Environmental adaptation			
**CheY**	Chemotaxis protein/ chemotaxis; signal transduction	Cytoplasm	A0A0F6C5H6_ECOLX
**FliC**	Flagellar filament structural protein/ cell motility	Extracellular	Q9L736_ECOLX
GroES	Co-chaperone GroES/ stress response	Cytoplasm	A0A0F6CCA2_ECOLX
SodA	Superoxide dismutase (Mn)/ superoxide metabolism; heat and acidic pH response	Cytoplasm	A0A0B1K7V5_ECOLX
TrxB	Thioredoxin reductase/ removal of superoxide radicals	Cytoplasm	A0A0F6C188_ECOLX
Respiration			
FdoH	Formate dehydrogenase O subunit beta/ anaerobic respiration; formate oxidation	Inner membrane	A0A0F6CBK4_ECOLX
NarZ	Nitrate reductase Z subunit alpha/ anaerobic respiration	Inner membrane	A0A0F3U4C6_ECOLX
Unknown			
CDCO157_2208	Hypothetical putative monooxygenase	-	A0A0F6C4V7_ECOLX
EC34880_0547	Hypothetical lipoprotein 16	-	L9IRW8_ECOLX
**C. LRF *In vitro*:**			
General function/ Protein name^1^	Specific function	Bacterial-cell localization	Accession number
Lipid and carbohydrate metabolism			
AccC	Biotin carboxylase/ fatty acid biosynthesis	Cytoplasm	A0A066REV0_ECOLX
Protein and nucleotide metabolism			
**Adk**	Adenylate kinase/ nucleotide biosynthesis	Cytoplasm	A0A0F6C005_ECOLX
**DeoB**	Phosphopentomutase/ nucleotide catabolism	Cytoplasm	A0A066SY18_ECOLX
HslU	ATPase component of the HslVU protease/ proteolysis	Cytoplasm	A0A066RBH1_ECOLX
Lon	Lon protease/ proteolysis	Cytoplasm	A0A0F6BZX1_ECOLX
Energy metabolism			
DkgA	Methylglyoxal reductase/ ketogluconate metabolism	Cytoplasm	A0A0F6C8Y9_ECOLX
GltA	Citrate synthase/ metabolism; electron transport chain	Cytoplasm	A0A0F6C0L6_ECOLX
PfkA	6-phosphofructokinase I/ glycolysis	Cytoplasm	A0A0J2D5M9_ECOLX
**Pgk**	Phosphoglycerate kinase/ gluconeogenesis	Cytoplasm	A0A0F6C8P8_ECOLX
Upp	Uracil phosphoribosyltransferase/ nucleoside metabolism	Cytoplasm	A0A0F6C7H7_ECOLX
SucD	Succinyl-CoA synthetase subunit alpha/ tricarboxylic acid cycle	Cytoplasm	A0A0F6C0M5_ECOLX
Transcription and translation			
**IhfA**	Integration host factor subunit alpha/ transcription regulator	Cytoplasm	A0A0F6C502_ECOLX
HupA	DNA-binding protein HU-alpha/ DNA transcription	Cytoplasm	A0A0F6CBV8_ECOLX
RplC	50S ribosomal subunit protein L3/ protein translation	Cytoplasm	A0A077K611_ECOLX
**RplO**	50S ribosomal subunit protein L15/ protein translation	Cytoplasm	A0A140RNX1_ECOLX
RpoB	RNA polymerase subunit beta/ DNA transcription	Cytoplasm	A0A0F6CBU4_ECOLX
**RpsC**	30S ribosomal subunit protein S3/ protein translation	Cytoplasm	A0A077K4Q6_ECOLX
**RpsD**	30S ribosomal subunit protein S4/ protein translation	Cytoplasm	A0A077K4W3_ECOLX
RpsK	30S ribosomal subunit protein S11/ protein translation	Cytoplasm	A0A077K6T2_ECOLX
**D. LRF *In vivo*:**			
General function/ Protein name^1^	Specific function	Bacterial-cell localization	Accession number
Lipid and carbohydrate metabolism			
BglA	6-phospho-beta-glucosidase A/ carbohydrate catabolism and metabolism	Cytoplasm	A0A0F6C8M6_ECOLX
**BglX**	Beta-D-glucoside glucohydrolase, periplasmic/ carbohydrate metabolism	Periplasm	A0A0F6C6I6_ECOLX
FabA	Beta-hydroxyacyl-acyl carrier protein dehydratase/isomerase/ fatty acid biosynthesis	Cytoplasm	A0A0F6C1F1_ECOLX
GmhA	D-sedoheptulose 7-phosphate isomerase/ carbohydrate biosynthesis	Cytoplasm	A0A0F6BZ89_ECOLX
HdhA	7-alpha-hydroxysteroid dehydrogenase/ lipid and bile acid catabolism	Cytoplasm	A0A0F6C4Q9_ECOLX
YeaE	Methylglyoxal reductase/ methylglyoxal catabolism	Cytoplasm	A0A0F6C8X8_ECOLX
Protein metabolism			
GlnA	Glutamine synthetase/ glutamine biosynthesis; nitrogen utilization	Cytoplasm	A0A0F6CBH7_ECOLX
GlyA	Serine hydroxymethyltransferase/ glycine biosynthesis	Cytoplasm	D3QN42_ECOCB
OmpT	Outer membrane protease VII (outer membrane protein 3b)/ proteolysis	Outer membrane	A0A0F6C344_ECOLX
SerC	Phosphoserine/phosphohydroxythreonine aminotransferase/ L-serine biosynthesis	Cytoplasm	A0A0F6C1A5_ECOLX
TdcF	Putative enamine/imine deaminase/ threonine metabolism	Cytoplasm	A0A0H3PZK0_ECO5C
Energy metabolism			
Agp	Glucose-1-phosphatase/ glucose catabolism	Periplasm	A0A0F3UBB5_ECOLX
**FbaB**	Fructose-bisphosphate aldolase class I/ glycolysis; gluconeogenesis	Cytoplasm	A0A037Y4V8_ECOLX
GldA	L-1, 2-propanediol dehydrogenase/glycerol dehydrogenase/ glycerol metabolism	Cytoplasm	A0A0F6CBQ9_ECOLX
**TktA**	Transketolase 1/ non-oxidative pentose phosphate pathway/metabolism	Cytoplasm	C3T0J6_ECOLX
UcpA	Putative oxidoreductase/ metabolism	Cytoplasm	A0A0D7CB02_ECOLX
YhcB	Cytochrome d ubiquinol oxidase subunit III/ oxidative phosphorylation; energy	Inner membrane	A0A0F6C9J7_ECOLX
Transcription and translation			
GlyS	Glycine-tRNA ligase subunit beta/ protein translation	Cytoplasm	A0A093EHH5_ECOLX
GreA	Transcription elongation factor GreA/ DNA transcription	Cytoplasm	K4UZX9_ECOLX
GrpE	Nucleotide exchange factor GrpE/ protein folding	Cytoplasm	A0A0E1IN68_ECOLX
NusA	Transcription termination/antitermination protein/ DNA transcription	Cytoplasm	A0A0F6C9E1_ECOLX
OsmY	Periplasmic chaperone OsmY/ protein folding	Periplasm	A0A0F6CCV9_ECOLX
PheS	Phenylalanine-tRNA ligase subunit alpha/ protein translation	Cytoplasm	A0A0F6C504_ECOLX
RpsF	30S ribosomal subunit protein S6/ protein translation	Cytoplasm	A0A0F6CCF6_ECOLX
Tsf	Protein chain elongation factor EF-Ts/ protein translation	Cytoplasm	A0A0F6BZ14_ECOLX
Transport			
**BtuB**	Cobalamin/cobinamide outer membrane transporter/ transport	Outer membrane	A0A0F6CBT1_ECOLX
**FadL**	Long-chain fatty acid outer membrane channel/bacteriophage T2 receptor/ lipid transport	Outer membrane	A0A0F6C745_ECOLX
FhuA	Ferrichrome outer membrane transporter/ phage receptor/ ion and siderophore transport	Outer membrane	A0A0F6BYZ6_ECOLX
ModA	Molybdate ABC transporter periplasmic binding protein/ molybdate transport	Periplasm	A0A0F3UDV0_ECOLX
OmpC	Outer membrane porin C/ transport	Outer membrane	A0A0F6C6S1_ECOLX
OmpF	Outer membrane porin F/ transport	Outer membrane	A0A0F3U779_ECOLX
SlbB	Soluble ligand binding β-grasp domain/ polysaccharide export	Outer membrane	A0A0F6C1P4_ECOLX
TolB	Tol-Pal system periplasmic protein TolB/ protein transport	Periplasm	A0A0F6C0P6_ECOLX
**TolC**	Outer membrane channel protein TolC/ transmembrane transport	Outer membrane	A0A0F6C915_ECOLX
**Tsx**	Nucleoside-specific channel-forming protein/ transmembrane transport	Outer membrane	A0A0D0NGK1_ECOLX
YbaT	Putative transporter/ amino acid transport	Plasma membrane	A0A061KFG2_ECOLX
Environmental adaptation			
FlgE	Flagellar hook protein/ cell motility	Cytoplasm	A0A0B0VWY9_ECOLX
**FliC**	Flagellar filament structural protein/ cell motility	Extracellular	Q9L736_ECOLX
FtnA	Ferritin iron storage protein/ iron uptake and storage; oxidative stress	Cytoplasm	A0A0F3TY56_ECOLX
GadA/B	Glutamate decarboxylase B/ acid resistance	Cytoplasm	A0A0F6C442_ECOLX
KatG	Hydroperoxidase (catalase-peroxidase) I/ oxidative stress response	Cytoplasm	A0A037YMJ1_ECOLX
OmpV	Outer membrane protein ompV/ scaffolding; bacterial adhesion	Outer membrane	A0A0F6C574_ECOLX
**OmpX**	Outer membrane protein X/ Bacterial adhesion	Outer membrane	A0A0F6C112_ECOLX
**SodA**	Superoxide dismutase (Mn)/ superoxide metabolism; heat and acidic pH response	Cytoplasm	A0A0F6C4U8_ECOLX
**TerD**	Tellurium resistance protein/ stress resistance	Inner membrane	A0A0F6C2A2_ECOLX
Tpx	Lipid hydroperoxidase peroxidase/ cell redox homeostasis	Cytoplasm	A0A0F6C3S0_ECOLX
YjbJ	Putative stress response protein/ osmotic shock response	Cytoplasm	A0A0F6CC06_ECOLX
Respiration			
NuoG	NADH: quinone oxidoreductase subunit G/ aerobic respiration	Inner membrane	A0A0F3UIJ5_ECOLX
Cell membrane			
**BamA**	Outer membrane protein assembly factor/ cell membrane assembly and integrity	Outer membrane	A0A0F6BZ21_ECOLX
**BamD**	Outer membrane protein assembly factor/ cell membrane assembly and integrity	Outer membrane	A0A0J2BAD4_ECOLX
**LptD**	Lipopolysaccharide assembly protein LptD/ lipopolysaccharide biosynthesis	Outer membrane	A0A0F6BYQ2_ECOLX
**SlyB**	Outer membrane lipoprotein slyB/ cell envelope biogenesis	Outer membrane	A0A0F6C4T3_ECOLX

^1^ Gene names in bold are of proteins also identified by iTRAQ analysis #2(reference-free analysis).

**(iii) Reference-free iTRAQ analysis #2 verified minimal association of differentially expressed proteins to specific O157 strains tested.** By comparing iTRAQ reporter ion intensities across the strains, without comparing to a reference strain proteome, a total of 667 and 473 proteins were identified as being differentially expressed, cumulatively, by the three O157 strains in the *in vitro* and *in vivo* growth conditions of MRF and LRF, respectively. This reference-free iTRAQ analysis was done to remove possible biases introduced by iTRAQ #1 analysis and verify if similar observations would be made. As with iTRAQ#1 analysis, iTRAQ #2 analysis determined more O157 proteins to be up-regulated in the rumen fluid from animals on the lactation diet (154 out of a total of 473 proteins; 33%) compared to those on the maintenance diet (81 out of a total of 667 proteins; 12%). In MRF, 25 proteins were more highly expressed in the *in vitro* condition and 56 were more highly expressed in the *in vivo* condition (S7 Table). In LRF, 43 proteins were more highly expressed in the *in vitro* condition and 111 were more highly expressed in the *in vivo* condition (S7B Table). Using these differentially expressed proteins, a GO term enrichment analysis was preformed to identify GO terms that were significantly enriched in each of the culture conditions within each rumen fluid (S8A–S8D Table).

**Fig 5:**
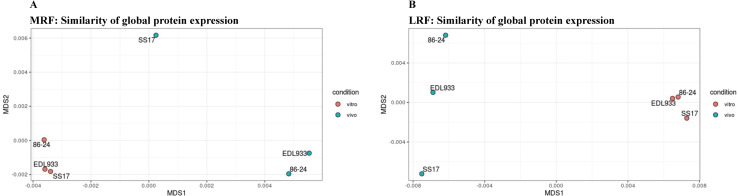
Multidimensional scaling plots generated with data from iTRAQ analysis #2 (reference-free). The plots depicting the similarity of global protein expression between O157 strains tested in (A) MRF or (B) LRF, *in vitro* and *in vivo*. This is based on Bray-Curtis dissimilarities generated from Scaffold normalized iTRAQ reporter intensities generated in reference-free iTRAQ analysis #2.

The normalized reporter intensities from this iTRAQ analysis #2 were then used to construct a Bray-Curtis dissimilarity matrix (multidimensional scaling or MDS) describing the similarities of the global protein expression between all samples within each rumen fluid (Fig 5). In MRF, the difference in global protein expression between the culture conditions was less evident (PERMANOVA *p* = 0.08, F = 3.7, R2 = 0.51) and no significant strain effect was detectable PERMANOVA *p* = 0.67) (Fig 5A, S9A Table). Within LRF, a significant difference in global protein expression patterns was detected between the two culture conditions, (PERMANOVA *p* = 0.05, F = 5.6, R2 = 0.572) but no significant difference was detected between the different strains (PERMANOVA *p* = 0.65) (Fig 5B, S9B Table). In both rumen fluids, protein expression patterns in the *in vivo* culture conditions appeared to have more variable protein expression patterns; this inference was derived from the dispersion distance plots as shown in Supplementary Fig 3. However, statistical testing failed to uncover sufficient evidence for this (PERMDISP2 *p* = 0.30 for both MRF and LRF).

Taken together these analyses indicated that differences in protein expression were mainly attributable to the growth conditions (*in vitro* and *in vivo*) and not the O157 strains. The proteome of O157 strain SS-17 appeared to have distinctive features compared to proteomes of 86–24 and EDL933, especially *in vitro* in LRF, although no statistical significance was observed. This distinction captured by both the MDS, and dispersion plots (Fig 5 and S4 Fig) appeared to reflect the survival patterns observed for SS-17 which varied from 86–24 and EDL933 (Figs 2 and 3).

### iBAQ and iTRAQ analyses identified similar O157 proteins associated with adaptive responses

Based on the iBAQ analysis, overall, 179 O157 proteins were determined to be unique to MRF and 11 unique to the LRF (Fig 4; S4B Table). The 179 O157 proteins uniquely expressed in MRF could be functionally assigned to lipid and carbohydrate metabolism, protein and nucleotide metabolism, energy metabolism, regulatory, transcription and translation, transport, cell integrity, cell division, environmental adaption, and respiration (S4B Table). Similarly, the 11 O157 proteins uniquely expressed in LRF could be assigned to lipid and carbohydrate metabolism (FolE, KdsA), protein metabolism (DmlA), energy metabolism (AceF, MaeA, SucC), regulatory (EngB), transcription and translation (FusA1, PrfC, RpsL), and environmental adaptation (OsmC) (S4B Table). No protein directly associated with virulence was identified in the O157 proteomes expressed in both MRF and LRF.

Proteins with similar functions were also identified by iTRAQ analysis #1. *In vitro*, the O157 strains highly expressed more proteins in LRF (19 proteins) than in MRF (12 proteins), relative to the reference 86–24 *in vitro* proteome, especially proteins associated with protein, nucleotide and energy metabolism, transcription, and translation (Table 1). This increased expression of proteins *in vitro* in LRF was associated mainly with the O157 strain SS-17 for 12/19 proteins (AccC, Adk, DeoB, HslU, Lon, DkgA, GltA, PfkA, Pgk, Upp, SucD, RpoB), even though SS-17 had the most suppressed growth (5-log reduction) in this growth condition. This was in contrast to *in vitro* in MRF where only 5/12 proteins were exclusively high in SS-17 (PckA, HupA, Hfq, RplU, RpsD) (Table 1, S6A and S6B Table, Fig 3). *In vivo*, a greater number of proteins were up-regulated in both LRF (52 proteins) and MRF (54 proteins), relative to the reference proteome. For the most part the proteins with increased expression *in vivo*, relative to the reference 86–24 *in vitro* proteome, could be associated with all three or at least two of the test strains except for 8/52 (GlnA, GlyA, YhcB, GlyS, FhuA, OmpC, OmpF, GadA/B), and 13/54 (AccC, RpiA, AcnB, CydA, GltA, SucC, SucD, Fur, ArgS, GlnS, RpoA, ValS, NarZ) proteins that were primarily high in SS-17 in LRF and MRF, respectively (Table 1, S6A and S6B Table). As observed with LRF *in vitro*, SS-17 demonstrated suppressed growth in MRF *in vivo* by 2-logs compared to other test bacteria (Fig 3). The correlation between growth suppression and increased expression of proteins of the lipid and carbohydrate metabolism, energy metabolism and transcription and translation is unclear but may indicate attempt at survival by stressed bacteria [69]. In the reference-free iTRAQ dataset, 17 and 22 proteins overlapped with proteins identified as having increased expression by iTRAQ analysis #1, in MRF and LRF, respectively. These are shown in bold in Table 1 under each of the MRF and LRF growth conditions. The increased expression of proteins in SS-17 may have also contributed to the distinctive profile in MDS and dispersion plots (Fig 5 and S3 Fig). Irrespective of the level of expression, no virulence protein was identified in the O157 proteomes, following *in vitro* or *in vivo* growth in either rumen fluids.

Interestingly GadA/B, associated with the glutamate-dependent acid resistance system, was the only acid-resistance related enzyme expressed in LRF, with increased expression *in vivo* (Table 1, S9A and S9B Table). With MRF, GadA/B was very minimally expressed under *in vitro* conditions only while expression of SpeB and PotD, associated with the arginine-dependent acid resistance system, was enhanced in all 3 O157 strains *in vivo* (Table 1, S6A and S6B Table).

## Discussion

In this study, we standardized a novel, non-terminal method that allows reuse of animals following *in vivo* studies with bacterial pathogens in the rumen of canulated cattle. The use of dialysis cartridges fitted with appropriate membranes ensured, (i) containment of bacteria being tested within the cartridges while exposing these bacteria to the rumen environment, (ii) simultaneous testing of multiple bacteria, and (iii) retrievability of these bacteria without euthanizing the animal.

Utilizing this method, survival, and protein expression of three O157 strains was evaluated along with a non-STEC *E*. *coli* Nal^R^ (#5735) as a comparative control, *in vitro* and *in vivo*, in ruminal fluids of contrasting compositions. Feeding dairy cows, a high fiber maintenance diet or a high energy-protein lactation diet resulted in rumen fluids with differing pH and VFA concentrations, termed MRF and LRF respectively. The pH ranged from 6.2–7.0 and total VFA concentrations from 109–141 μM/ml in MRF while LRF had a ruminal pH ranging between 5.2–6.1, and total VFA of 125–219 μM/ml (Table 1A and 1B in Fig 2 and Table 2A and 2B in Fig 3). An increase in the total VFA concentrations was observed post-incubation with test bacteria under *in vitro* growth conditions, in MRF (VFA of 167–181 μM/ml) and LRF (VFA of 215–260 μM/ml) (Table 1A in Fig 2 and Table 2A in Fig 3). As previously reported [28] this increase in *in vitro* VFA may have been due to breakdown of the limited nutrients by all microbes in the rumen fluid in the absence of a dynamic turn-over seen *in vivo* in the rumen of a consistently fed animal demonstrating the limitations in reproducing *in vivo* rumen conditions *in vitro*. Key differences were observed in the survival of the bacteria tested in either rumen fluids, under both growth conditions. The O157 strains 8624 and EDL933 had similar survival patterns, *in vitro* and *in vivo*, in both rumen fluids (Figs 1 and 2). In contrast, the O157 strain SS17 and *E*. *coli* Nal^R^ (#5735) had the most varied survival patterns. *E*. *coli* Nal^R^ (#5735) and O157 strain SS17 demonstrated the highest decrease in viable counts *in vitro* in MRF (3-log decrease) and LRF (5-log decrease), respectively; the opposite was observed *in vivo* with a 3-log decrease for *E*. *coli* Nal^R^ (#5735) in LRF and a 2-log decrease in MRF for SS17 (Figs 1 and 2, S2 and S3 Tables). These observations clearly indicate the need for reliable methods for studying bacteria *in vivo* in the animal as the environmental cues seen within cannot be fully reproduced *in vitro* and bacterial strains can respond differently to such cues. Lack of ease in conducting rumen site-specific studies has resulted in limited *in vivo* in animals compared to *in vitro* studies [27,31–35] which can be circumvented using our novel method of analysis.

Diet, growth conditions and challenge with O157 strains has been shown to influence ruminal pH and VFA concentrations. For instance, an average ruminal pH of 5.98 over 29 days post-O157 strain 86–24 challenge was observed *in vivo* in cattle fed a grain diet comprising of 90% corn and 10% alfalfa [32], while an *in vitro* study displayed a decrease of 1.4 pH units from 7.25 prior to incubation to 5.85 twenty-four hours post-O157 strain EDL933 inoculation in rumen fluid from cattle fed a mixed diet of 80% hay and 20% concentrate [70]. Alongside pH, ruminal VFA concentration was analyzed in the latter study. Prior to incubation, VFA concentration was measured at 115.78 mM, decreasing at hour 6 to 90.8 mM, and increasing to 129.53 mM after 24 hours, with a high acetate concentration of 84.51 mM [71]. Our previous *in vitro* study corroborates this result, as acetate accounted for the majority of VFAs measured in unfiltered, filtered, and depleted rumen fluid, prepared from rumen fluid from animals fed the high fiber maintenance diet, at 62–72% of VFAs measured, with propionate and butyrate concentrations ranging from 13–18% and 6–13%, respectively [28]. In this study, irrespective of the growth condition, VFA concentrations were higher in LRF compared to MRF, derived from animals fed a high protein versus high fiber diet, respectively. This high VFA concentration may have stemmed from increased fermentation in LRF which in turn contributed to its acidic pH; percent concentration of acetate was slightly higher in MRF while butyrate was higher in LRF (Table 1A and 1B in Fig 2 and Table 2A and 2B in Fig 3).

O157 strains can express specific genes/proteins, to prolong survival in certain environmental conditions as influenced by VFA concentrations and pH in the rumen fluid. In our study, we used riBAQ analysis of the proteomics data to determine differences in proteins expressed between the contrasting rumen fluids. This was done since the proteomics data was generated in two different LC-MS/MS runs with each type of rumen fluid represented only on one run. The riBAQ analysis of the proteomics data, based on relative abundance, was useful in identifying proteins uniquely expressed in LRF (11) versus MRF (179), although caution is required when comparing protein relative abundances between different rumen fluids as compositional effects could led to some erroneous conclusions (more proteins under the limit of detection in LRF could have led to an artificial inflation of the proportions of the detected proteins). *In silico* analysis of the iTRAQ dataset using reporter intensities attributed differential expression of proteins mainly to culture conditions rather than individual bacterial strains being tested, as evidenced in the MDS plot (Fig 5), irrespective of the rumen fluid. The reference-based analysis of the iTRAQ dataset further identified proteins with increased expression *in vitro* and *in vivo* relative to the 86–24 *in-vitro* proteome, in both LRF and MRF. Under *in vitro* growth condition expression of more proteins was increased in LRF (19 proteins) than MRF (12 proteins), compared to *in vivo* growth conditions where greater number of proteins were up-regulated in both LRF (52 proteins) and MRF (54 proteins). Interestingly, SS17 seemed to contribute most towards some of the differentially expressed O157 proteins *in vitro* in LRF, and *in vivo* in MRF. While our multivariate statistical tests did not detect robust strain differences, SS17 exhibited some indications of strain level differences with altered growth characteristics and indications of altered protein expression. This distinctive feature of the SS-17 proteome was identified by the MDS, and dispersion plots based on reference-free analysis of the iTRAQ data as well. SS17 demonstrated greater growth suppression *in vitro* in LRF, and *in vivo* in MRF, and hence the increased protein expression may correlate with a possible stress response [69].

Transcriptome analyses of O157 during growth in the rumen have revealed several pathways, genes, and proteins that are differentially expressed. Hughes *et al*. found that SdiA-AHL (acyl-homoserine lactones) sensing alters 49 EHEC genes while in the rumen, including those involved in acid resistance (*gad*) and the locus of enterocyte effacement (LEE) [32], with similar results observed by Nguyen *et al*. when using AHL synthase gene *yenI* [72]. Rumen AHLs sensed by SdiA directly repress LEE gene transcription, but indirectly induces *gad* acid resistance genes to increase EHEC’s chance of survival in acidic environments like the rumen [32]. A 2018 transcriptome study conducted by Segura *et al*. found that O157 strain EDL933 differentially expresses 617 genes in the bovine rumen in comparison to minimal media [70]. Upregulation of genes within carbohydrate transport (*xylA/F*, *fucA/O*, *rbsACD*, *cmtB*), amino acid transport (*dctA*, *tnaA*, *tdcD/E*, *asnA*), energy production and conversion (*appBC*, *dmsABC*, *napAH*, *narGHIJ*, *nrfEFG*), and aerobic respiration (*fdoHI*, *appBC glpD*) were observed. Ethanolamine is an important nitrogen source and growth aid for bacteria in the digesta and may also act as a signal for gene regulation while in the rumen, as observed by induction of assimilation and transport genes (*eut*) in the rumen content [70]. Our previous work also showed expression of several O157 proteins involved in cell structure (OmpA/W, BamA/D, LppC, and AsmA), metabolism (LysU, HyC, TnaA, and LamB), chemotaxis (GgbP, Trg), adherence (AidA-1, EspP, and CsgB/G), and stress responses (KatG, HchA, MoaB, and the Arg acid resistance system) but none associated with O157 virulence *in vitro*, in the bovine rumen fluid [28]. This led to the conclusion that rumen-specific protein expression allows O157 to adapt and survive within the rumen in order to move throughout and colonize the bovine gastrointestinal tract.

Similarly, in our present study, we observed numerous O157 proteins belonging to nine general function categories were upregulated in the rumen fluid from cows fed the maintenance (MRF) or lactation (LRF) diets. General protein functions included: metabolism (lipid and carbohydrate, amino acid and protein, energy), regulation, translation and transcription, transport, cell membrane synthesis, environmental adaptation, and respiration (Table 1). Proteins expressed *in vivo* irrespective of diet type with a role in metabolism (YhcB, OmpT), protein translation (RpsF), transport (FhuA, ModA), or environmental adaptation (FliC, SodA) were also identified in other proteomic and transcriptomic rumen fluid studies, described hereafter. An *in vitro* model by Segura *et al*. identified transport proteins ModA and FeoA [70] expressed by O157 strain EDL933, in rumen fluid extracted at slaughter from bulls fed a mixed diet of 80% hay and 20% concentrate. In addition to the ModA and FeoA transport proteins, proteins involved in metabolism (MalK, GltA, PckA, SucC/D), or respiration (SdhB) were also upregulated, supporting our findings. Lastly, SpeB, RpsF, and FliC were identified in our previous proteomics work as well, using cattle on a low protein maintenance diet consisting of 25% fiber and 10% protein [28]. Other proteins from the current study were also identified in our earlier work, spanning each general function category- except for regulation- including DegQ, FbaB, OmpC, FtnA, RpoA, Tig, RbsB, BamA/D, TerD, and FdoH, in different preparations of the rumen fluid [28].

Three O157 acid-resistance systems have been identified thus far: glucose-repressed or oxidative, glutamate-dependent, and arginine-dependent [16,73–75]. The glucose-repressed system is activated during stationary phase via RpoS and cAMP receptor protein and repressed with the addition of glucose. The glutamate and arginine systems utilize either GadA/B or AdiA decarboxylases to convert glutamate or arginine to gamma-aminobutyric acid (GABA) or agmatine, respectively, consuming excess protons in the process. Agmatine is either exported via the arginine-agmatine antiporter (*aidC*) or converted (*speB*) to putresceine as part of the polyamine biosynthetic pathway and putresceine transported out of the cell via the ABC transporter encoded by *potD* [73,74]. GABA is transported out of the cell via the antiporter encoded by *gadC*. The glutamate system seems to provide the most protection against acidic environments (below pH 6) [16,73,74]. In fact, in cattle fed a high grain diet of 90% corn and 10% alfalfa and challenged with O157 strain 86–24, an upregulation of *gad* transcripts was observed in rumen fluid post-challenge, activated by acyl-homoserine lactones (AHLs) and SdiA [27,32]. In a similar study, *gad* gene expression was induced *in vitro* by O157 strain 86–24 engineered to express AHL synthase, *yenI*, to mimic constant AHL exposure [72]. Other acid resistance systems are activated between pH 5.5–6.9 and used more often than the glutamate system to counter the deleterious effects of protons [73,74]. In our study, GadA/B was the only acid-resistance related enzyme expressed in the acidic LRF with increased expression *in vivo* (Table 1). In MRF on the other hand, as observed in our previous study, minimal expression of GadA/B was observed *in vitro* only while SpeB and PotD expression was enhanced in all 3 O157 strains tested *in vivo* (Table 1).

None of the proteins expressed in LRF and MRF were associated with O157 virulence, irrespective of the *in vitro* and *in vivo* growth conditions or the O157 strain tested. Using the reference-based iTRAQ analysis #1, 66 and 71 proteins were determined to have increased expression, in *in vitro* and *in vivo* conditions combined, in MRF and LRF, respectively (Table 1, Fig 4). Of these, 17/66 and 22/71 proteins were identified as being up-regulated in either *in vitro* or *in vivo* conditions by reference free iTRAQ analysis #2 as well (Table 1 shown in bold) and are of interest along with the acid resistance mechanisms for further evaluation in therapeutic modalities. In conclusion, this study highlights a novel method for introducing and retrieving bacteria from the rumen of animals for *in vivo* evaluations and the application of different proteomics data analysis methods to derive useful protein expression data. The reusable animal model is very cost-effective as multiple studies with access to the internal rumen environment that cannot be fully reproduced *in vitro* can be conducted, with minimal number of animals. The observations made reinforced that the maintenance and lactation diets have differing influences on the rumen environment which in turn affects O157 survival and protein expression. The O157 proteins uniquely expressed *in vivo* in the rumen vary from those expressed *in vitro* in the rumen fluid but taken together further support our previous conclusions that O157 induces expression of proteins from a broad range of functions to adapt and survive the diverse physiological and microbiome conditions within the bovine rumen. Given that the expression of these proteins is mainly influenced by the animals’ diet and not O157 strain type, preharvest control modalities could be developed with these proteins to target O157 across strains in cattle at all stages of husbandry. Additional experiments are being planned to study the influence of the rumen environment, under similar dietary conditions, on O157, non-O157 and commensal *E*. *coli* gene expression at various time-points post-exposure, to obtain a dynamic and comparative profile of the adaptive responses of these bacteria.

## Supporting information

S1 Fig Product labels for ‘Steakmaker’ and ‘Lactation Premix’ used in the animal diets. (TIF)

S2 Fig Experimental design. (TIF)

S3 Fig Total and relative iBAQ value plots. (TIF)

S4 Fig Dispersion plots. (TIF)

S1 Table Recovery of *E*. *coli* Nal^R^ (#5735) in dialysis PVDF tubings versus cartridges exposed to MRF in *in vitro* and *in vivo* pilot experiments. (DOCX)

S2 Table Recovery of bacteria from cartridges exposed to MRF in *in vitro* and *in vivo*. (DOCX)

S3 Table Recovery of bacteria from cartridges exposed to LRF in *in vitro* and *in vivo*. (DOCX)

S4 Table **A:** iBAQ and riBAQ values of proteins expressed by O157 strains in MRF and LRF. **B:** Proteins uniquely expressed by O157 strains in MRF and LRF, irrespective of the in vitro/in vivo growth conditions, identified using iBAQ. **C:** Proteins more highly expressed by O157 strains in MRF; based on riBAQ values of L2FC < -1 only. **D:** Proteins more highly expressed by O157 strains in LRF; based on riBAQ values of L2FC > 1 only.
(ZIP)

S5 Table **A:** GO terms of O157 proteins enriched in MRF; based on riBAQ values of L2FC < -1 and Fisher *p* values < 0.1. **B:** GO terms of O157 proteins enriched in LRF; based on riBAQ values of L2FC >1 and Fisher *p* values < 0.1.
(ZIP)

S6 Table **A:** iTRAQ quantitative samples view report of proteins differentially expressed by O157 strains in MRF, in comparison to the 86–24 *in vitro* proteome (reference-based analysis). **B:** iTRAQ quantitative samples view report of proteins differentially expressed by O157 strains in LRF, in comparison to the 86–24 *in vitro* proteome (reference-based analysis).
(ZIP)

S7 Table **A.** O157 proteins significantly different between the *in vitro* and *in vivo* culture conditions in MRF determined by using reference-free analysis of iTRAQ data. **B.** O157 proteins significantly different between the *in vitro* and *in vivo* culture conditions in LRF determined by using reference-free analysis of iTRAQ data.
(ZIP)

S8 Table **A.** GO terms of O157 proteins enriched in the *in vitro* growth condition, in MRF; based on reference-free, Scaffold normalized iTRAQ intensities resulting in proteins with L2FC values < -0.5 and Fisher *p* values < 0.1 only. **B.** GO terms of O157 proteins enriched in the *in vivo* growth condition, in MRF; based on reference-free, Scaffold normalized iTRAQ intensities resulting in proteins with L2FC values > 0.5 and Fisher *p* values < 0.1. **C.** GO terms of O157 proteins enriched in the *in vitro* growth condition, in LRF; based on reference-free, Scaffold normalized iTRAQ intensities resulting in proteins with L2FC values < -0.5 and Fisher *p* values < 0.1 only. **D.** GO terms of O157 proteins enriched in the *in vivo* growth condition, in LRF; based on reference-free, Scaffold normalized iTRAQ intensities resulting in proteins with L2FC values > 0.5 and Fisher *p* values < 0.1.
(ZIP)

S9 Table **A.** PERMANOVA results detailing strain and condition effects within MRF. Based on Bray-Curtis dissimilarities generated from reference-free, Scaffold normalized iTRAQ intensities. **B.** PERMANOVA results detailing strain and condition effects within LRF. Based on Bray-Curtis dissimilarities generated from reference-free, Scaffold normalized iTRAQ intensities.
(RAR)

S1 Text NCBI_Ecoli_used_for_reference. (PDF)

S2 Text MQ_O157_parameters. (PDF)

S3 Text (DS_Store)

## References

[1] Tack DM RL, Griffin PM, et al. Preliminary Incidence and Trends of Infections with Pathogens Transmitted Commonly Through Food—Foodborne Diseases Active Surveillance Network, 10 U.S. Sites, 2016–2019. MMWR Morb Mortal Wkly Rep 2020;69:509–14. doi: 10.15585/mmwr.mm6917a1 32352955 PMC7206985

[2] Scallan E, Griffin PM, Angulo FJ, Tauxe RV, Hoekstra RM. Foodborne Illness Acquired in the United States—Unspecified Agents. Emerging Infectious Diseases. 2011;17(1):16–22. doi: 10.3201/eid1701.091101p2 21192849 PMC3204615

[3] Majowicz SE, Scallan E, Jones-Bitton A, Sargeant JM, Stapleton J, Angulo FJ, et al. Global incidence of human Shiga toxin-producing *Escherichia coli* infections and deaths: a systematic review and knowledge synthesis. Foodborne Pathog Dis. 2014;11(6):447–55. doi: 10.1089/fpd.2013.1704 24750096 PMC4607253

[4] Kaper JB, O’Brien AD. *Escherichia coli* O157:H7 and Other Shiga Toxin-Producing E. coli Strains. Washington, DC: ASM Press; 1998.

[5] Paton JC, Paton AW. Pathogenesis and Diagnosis of Shiga Toxin-Producing *Escherichia coli* Infections. Clin Microbiol Rev. 1998;11:450–79. doi: 10.1128/CMR.11.3.450 9665978 PMC88891

[6] Riley LW, Memis RS, Helgerson SD, McGee HB, Wells JG, Davis BR, et al. Hemorrhagic colitis associated with a rare *Escherichia coli* serotype. New Eng J Med. 1983;308(12):681–5. doi: 10.1056/NEJM198303243081203 6338386

[7] Mead PS, Griffin PM. *Escherichia coli* O157:H7. Lancet. 1998;352:1207–12. doi: 10.1016/S0140-6736(98)01267-7 9777854

[8] Batz MB, Hoffmann S, Morris JG Jr. Ranking the disease burden of 14 pathogens in food sources in the United States using attribution data from outbreak investigations and expert elicitation. J Food Prot. 2012;75(7):1278–91. doi: 10.4315/0362-028X.JFP-11-418 22980012

[9] Bell BP, Goldoft M, Griffin PM, Davis MA, Gordon DC, Tarr PI, et al. A multistate outbreak of *Escherichia coli* O157:H7-associated bloody diarrhea and hemolytic uremic syndrome from hamburgers. JAMA. 1994;272(17):1349–53. 7933395

[10] Mayer CL, Leibowitz CS, Kurosawa S, Stearns-Kurosawa DJ. Shiga toxins and the pathophysiology of hemolytic uremic syndrome in humans and animals. Toxins (Basel). 2012;4(11):1261–87. doi: 10.3390/toxins4111261 23202315 PMC3509707

[11] Rivas M, Chinen I, Miliwebsky E, Masana M. Risk Factors for Shiga Toxin-Producing *Escherichia coli*-Associated Human Diseases. Microbiol Spectr. 2014;2(5). doi: 10.1128/microbiolspec.EHEC-0002-2013 26104362

[12] Kaper JB, Nataro JP, Mobley HL. Pathogenic *Escherichia coli*. Nat Rev Microbiol. 2004;2(2):123–40. doi: 10.1038/nrmicro818 15040260

[13] Ray PE, Liu X. Pathogenesis of Shiga toxin-induced hemolytic uremic syndrome. Pediatr Nephrol. 2001;16:823–39. doi: 10.1007/s004670100660 11605791

[14] Persad AK, LeJeune JT. Animal Reservoirs of Shiga Toxin-Producing *Escherichia coli*. Microbiol Spectr. 2014;2(4):EHEC-0027-2014. doi: 10.1128/microbiolspec.EHEC-0027-2014 26104194

[15] Kudva IT, Hovde CJ. *Escherichia coli* O157:H7 in Ruminants-A Review. Pandalai SG, editor. Trivandrum India: Research Signpost; 1998. 665–92 p.

[16] Nguyen Y, Sperandio V. Enterohemorrhagic *E*. *coli* (EHEC) pathogenesis. Front Cell Infect Microbiol. 2012;2:90. doi: 10.3389/fcimb.2012.00090 22919681 PMC3417627

[17] Naylor SW, Low JC, Besser TE, Mahajan A, Gunn GJ, Pearce MC, et al. Lymphoid follicle-dense mucosa at the terminal rectum is the principal site of colonization of enterohemorrhagic *Escherichia coli* O157:H7 in the bovine host. Infect Immun. 2003;71(3):1505–12. doi: 10.1128/IAI.71.3.1505-1512.2003 12595469 PMC148874

[18] Keen JE, Laegreid WW, Chitko-McKown CG, Durso LM, Bono JL. Distribution of Shiga-Toxigenic *Escherichia coli* O157 in the Gastrointestinal Tract of Naturally O157-Shedding Cattle at Necropsy. Appl Environ Microbiol. 2010;76(15):5278–81. doi: 10.1128/AEM.00400-10 20543036 PMC2916454

[19] Wells JE, Berry ED, Kim M, Bono JL, Oliver WT, Kalchayanand N, et al. Determination of gastrointestinal tract colonization sites from feedlot cattle transiently shedding or super-shedding *Escherichia coli* O157:H7 at harvest. J Appl Microbiol. 2020;129(5):1419–26. doi: 10.1111/jam.14684 32350973

[20] Fegan N, Vanderlinde P, Higgs G, Desmarchelier P. The prevalence and concentration of *Escherichia coli* O157 in faeces of cattle from different production systems at slaughter. J Appl Microbiol. 2004;97(2):362–70. doi: 10.1111/j.1365-2672.2004.02300.x 15239703

[21] Widiasih DA, Ido N, Omoe K, Sugii S, Shinagawa K. Duration and magnitude of faecal shedding of Shiga toxin-producing *Escherichia coli* from naturally infected cattle. Epidemiol Infect. 2004;132(1):67–75. doi: 10.1017/s0950268803001468 14979592 PMC2870080

[22] Lim JY, Li J, Sheng H, Besser TE, Potter K, Hovde CJ. *Escherichia coli* O157:H7 colonization at the rectoanal junction of long-duration culture-positive cattle. Appl Environ Microbiol. 2007;73(4):1380–2. doi: 10.1128/AEM.02242-06 17189448 PMC1828644

[23] Cobbold RN, Hancock DD, Rice DH, Berg J, Stilborn R, Hovde CJ, et al. Rectoanal junction colonization of feedlot cattle by *Escherichia coli* O157:H7 and its association with supershedders and excretion dynamics. Appl Environ Microbiol. 2007;73(5):1563–8. doi: 10.1128/AEM.01742-06 17220263 PMC1828767

[24] Omisakin F, MacRae M, Ogden ID, Strachan NJ. Concentration and prevalence of *Escherichia coli* O157 in cattle feces at slaughter. Appl Environ Microbiol. 2003;69(5):2444–7. doi: 10.1128/AEM.69.5.2444-2447.2003 12732509 PMC154535

[25] Matthews L, Low JC, Gally DL, Pearce MC, Mellor DJ, Heesterbeek JA, et al. Heterogeneous shedding of *Escherichia coli* O157 in cattle and its implications for control. Proc Natl Acad Sci U S A. 2006;103(3):547–52. doi: 10.1073/pnas.0503776103 16407143 PMC1325964

[26] Keen JE, Laegreid WW, Chitko-McKown CG, Durso LM, Bono JL. Distribution of Shiga-toxigenic *Escherichia coli* O157 in the gastrointestinal tract of naturally O157-shedding cattle at necropsy. Appl Environ Microbiol. 2010;76(15):5278–81. doi: 10.1128/AEM.00400-10 20543036 PMC2916454

[27] Sperandio V. SdiA sensing of acyl-homoserine lactones by enterohemorrhagic E. coli (EHEC) serotype O157:H7 in the bovine rumen. Gut Microbes. 2010;1(6):432–5. doi: 10.4161/gmic.1.6.14177 21468228 PMC3056111

[28] Kudva IT, Stanton TB, Lippolis JD. The Escherichia coli O157:H7 bovine rumen fluid proteome reflects adaptive bacterial responses. BMC Microbiology. 2014;14(48). doi: 10.1186/1471-2180-14-48 24559513 PMC3936929

[29] Lima FS, Oikonomou G, Lima SF, Bicalho ML, Ganda EK, Filho JC, et al. Prepartum and postpartum rumen fluid microbiomes: characterization and correlation with production traits in dairy cows. Appl Environ Microbiol. 2015;81(4):1327–37. doi: 10.1128/AEM.03138-14 25501481 PMC4309715

[30] Auffret MD, Dewhurst RJ, Duthie CA, Rooke JA, John Wallace R, Freeman TC, et al. The rumen microbiome as a reservoir of antimicrobial resistance and pathogenicity genes is directly affected by diet in beef cattle. Microbiome. 2017;5(1):159. doi: 10.1186/s40168-017-0378-z 29228991 PMC5725880

[31] Zhou M, Hünerberg M, Chen Y, Reuter T, McAllister TA, Evans F, et al. Air-Dried Brown Seaweed, Ascophyllum nodosum, Alters the Rumen Microbiome in a Manner That Changes Rumen Fermentation Profiles and Lowers the Prevalence of Foodborne Pathogens. mSphere 2018;3(1): e00017–18; doi: 10.1128/mSphere.00017-18 29404417 PMC5793039

[32] Hughes DT, Terekhova DA, Liou L, Hovde CJ, Sahl JW, Patankar AV, et al. Chemical sensing in mammalian host-bacterial commensal associations. Proc Natl Acad Sci U S A. 2010;107(21):9831–6. doi: 10.1073/pnas.1002551107 20457895 PMC2906910

[33] Sheng H, Nguyen YN, Hovde CJ, Sperandio V. SdiA aids enterohemorrhagic *Escherichia coli* carriage by cattle fed a forage or grain diet. Infect Immun. 2013;81(9):3472–8. doi: 10.1128/IAI.00702-13 23836826 PMC3754220

[34] Bertin Y, Habouzit C, Duniere L, Laurier M, Durand A, Duchez D, et al. Lactobacillus reuteri suppresses *E*. *coli* O157:H7 in bovine ruminal fluid: Toward a pre-slaughter strategy to improve food safety? PLoS One. 2017;12(11):e0187229. doi: 10.1371/journal.pone.0187229 29091926 PMC5665532

[35] Free AL, Duoss HA, Bergeron LV, Shields-Menard SA, Ward E, Callaway TR, et al. Survival of O157:H7 and non-O157 serogroups of *Escherichia coli i*n bovine rumen fluid and bile salts. Foodborne Pathog Dis. 2012;9(11):1010–4. doi: 10.1089/fpd.2012.1208 22957973 PMC3540925

[36] Tarr PI, Neill MA, Clausen CR, Newland JW, Neill RJ, Moseley SL. Genotypic variation in pathogenic *Escherichia coli* O157:H7 isolated from patients in Washington, 1984–1987. J Infect Dis. 1989;159(2):344–7. doi: 10.1093/infdis/159.2.344 2644374

[37] O’Brien AD, Melton AR, Schmitt CK, McKee ML, Batts ML, Griffin DE. Profile of *Escherichia coli* O157:H7 pathogen responsible for hamburger-borne outbreak of hemorrhagic colitis and hemolytic uremic syndrome in Washington. J Clin Microbiol. 1993;31(10):2799–801. doi: 10.1128/jcm.31.10.2799-2801.1993 8253989 PMC266020

[38] Cote R, Katani R, Moreau MR, Kudva IT, Arthur TM, DebRoy C, et al. Comparative analysis of super-shedder strains of *Escherichia coli* O157:H7 reveals distinctive genomic features and a strongly aggregative adherent phenotype on bovine rectoanal junction squamous epithelial cells. PLoS One. 2015;10(2):e0116743. doi: 10.1371/journal.pone.0116743 25664460 PMC4321836

[39] Moran J. How the Rumen Works. Tropical dairy farming: feeding management for small holder dairy farmers in the humid tropics: Landlinks Press; 2005. p. 312.

[40] Erickson PS, Kalscheur KF. Nutrition and feeding of dairy cattle. Animal Agriculture 2020. p. 157–80.

[41] Kudva IT, Hatfield PG, Hovde CJ. Effect of diet on the shedding of *Escherichia coli* O157:H7 in a sheep model. Appl Environ Microbiol. 1995;61(4):1363–70. doi: 10.1128/aem.61.4.1363-1370.1995 7747956 PMC167392

[42] Kudva IT, Hatfield PG, Hovde CJ. Characterization of *Escherichia coli* O157:H7 and other Shiga toxin-producing *E*. *coli* serotypes isolated from sheep. J Clin Microbiol. 1997;35(4):892–9. doi: 10.1128/jcm.35.4.892-899.1997 9157149 PMC229697

[43] Kudva IT, Hunt CW, Williams CJ, Nance UM, Hovde CJ. Evaluation of dietary influences on *Escherichia coli* O157:H7 shedding by sheep. Appl Environ Microbiol. 1997;63(10):3878–86. doi: 10.1128/aem.63.10.3878-3886.1997 9327551 PMC168697

[44] Van Gylswyk NO, Wejdemar K, Kulander K. Comparative Growth Rates of Various Rumen Bacteria in Clarified Rumen Fluid from Cows and Sheep Fed Different Diets. App Environ Microbiol. 1992;58:99–105. 10.1128/aem.58.1.99-105.1992 PMC195178 1539997

[45] Nikkhah A. Bioscience of ruminant intake evolution: feeding time models. Advances in Bioscience and Biotechnology. 2011;02(04):271–4.

[46] Salanitro JP, Muirhead PA. Quantitative Method for the Gas Chromatographic Analysis of Short-Chain Monocarboxylic and Dicarboxylic Acids in Fermentation Media. App Microbiol. 1975;29:374–81. doi: 10.1128/am.29.3.374-381.1975 1167776 PMC186983

[47] Lambert MA, Moss CW. Preparation and analysis of the butyl esters of short-chain volatile and non-volatile fatty acids. J Chromatograph 1972;74:335–8. 10.1016/s0021-9673(01)86164-1 4645673

[48] C.M.B. M. Anatomy and Physiology of the Rumen. In: Millen D., De Beni Arrigoni M., LP R., editors. Rumenology: Springer, Cham.; 2016.

[49] Anderson KL, Whitlock JE, Harwood VJ. Persistence and differential survival of fecal indicator bacteria in subtropical waters and sediments. Appl Environ Microbiol. 2005;71(6):3041–8. doi: 10.1128/AEM.71.6.3041-3048.2005 15933000 PMC1151827

[50] Welch JG. Rumination, particle size and passage from the rumen. J Animal Sci. 1982;54:885–94.

[51] Kudva IT, Krastins B, Sheng H, Griffin RW, Sarracino DA, Tarr PI, et al. Proteomics-based expression library screening (PELS): a novel method for rapidly defining microbial immunoproteomes. Mol Cell Proteomics. 2006;5(8):1514–9. doi: 10.1074/mcp.T600013-MCP200 16737953 PMC2754196

[52] Lippolis JD, Bayles DO, Reinhardt TA. Proteomic changes in *Escherichia coli* when grown in fresh milk versus laboratory media. J Prot Res 2009;8:149–58. doi: 10.1021/pr800458v 19053534

[53] Tyanova S, Temu T, Cox J. The MaxQuant computational platform for mass spectrometry-based shotgun proteomics,. Nat Protocols. 2016;11:2301–19. doi: 10.1038/nprot.2016.136 27809316

[54] Fu L, Niu B, Zhu Z, Wu S, Li W. CD-HIT: accelerated for clustering the next-generation sequencing data. Bioinformatics. 2012;28(23):3150–2. doi: 10.1093/bioinformatics/bts565 23060610 PMC3516142

[55] UniProt C. UniProt: the universal protein knowledgebase in 2021. Nucleic Acids Res. 2021;49(D1):D480–D9. doi: 10.1093/nar/gkaa1100 33237286 PMC7778908

[56] Shin JB, Krey JF, Hassan A, Metlagel Z, Tauscher AN, Pagana JM, et al. Molecular architecture of the chick vestibular hair bundle. Nat Neurosci. 2013;16(3):365–74. doi: 10.1038/nn.3312 23334578 PMC3581746

[57] Keller A, Nesvizhskii AI, Kolker E, Aebersold R. Empirical Statistical Model To Estimate the Accuracy of Peptide Identifications Made by MS/MS and Database Search. Anal Chem 2002;74:5383–92. doi: 10.1021/ac025747h 12403597

[58] Nesvizhskii AI, Keller A, Kolker E, Aebersold R. A Statistical Model for Identifying Proteins by Tandem Mass Spectrometry. Anal Chem 2003;75:4646–58. doi: 10.1021/ac0341261 14632076

[59] Shadforth IP, Dunkley TP, Lilley KS, Bessant C. i-Tracker: for quantitative proteomics using iTRAQ. BMC Genomics. 2005;6:145. doi: 10.1186/1471-2164-6-145 16242023 PMC1276793

[60] Oberg AL, Mahoney DW, Eckel-Passow JE, Malone CJ, Wolfinger RD, Hill EG, et al. Statistical analysis of relative labeled mass spectrometry data from complex samples using ANOVA. J Proteome Res. 2008;7(1):225–33. doi: 10.1021/pr700734f 18173221 PMC2528956

[61] Yu NY, Wagner JR, Laird MR, Melli G, Rey S, Lo R, et al. PSORTb 3.0: improved protein subcellular localization prediction with refined localization subcategories and predictive capabilities for all prokaryotes. Bioinformatics. 2010;26(13):1608–15. doi: 10.1093/bioinformatics/btq249 20472543 PMC2887053

[62] Jones P, Binns D, Chang HY, Fraser M, Li W, McAnulla C, et al. InterProScan 5: genome-scale protein function classification. Bioinformatics. 2014;30(9):1236–40. doi: 10.1093/bioinformatics/btu031 24451626 PMC3998142

[63] R, Core, Team. R: A language and environment for statistical computing. R Foundation for statistical Computing, Vienna, Austria URL https://wwwR-projectorg/. 2020.

[64] Alexa A, Rahnenfuhrer J. topGO: Enrichment Analysis for Gene Ontology. R package version 2.42.0. 2020.

[65] Ashburner M, Ball CA, Blake JA, Botstein D, al. e. Gene Ontology: tool for the unification of biology. Nat Genet. 2000;25:25–9. doi: 10.1038/75556 10802651 PMC3037419

[66] Consortium. TGO. The Gene Ontology resource: enriching a GOld mine. Nucleic Acids Res. 2021;49:D325–D34. doi: 10.1093/nar/gkaa1113 33290552 PMC7779012

[67] Oksanen J, Guillaume Blanchet F, Friendly M, Kindt R, Legendre P, McGlinn D, et al. Vegan: Community Ecology Package. R package version 25–7 https://CRANR-projectorg/package=vegan. 2020.

[68] Krey JF, Wilmarth PA, Shin JB, Klimek J, Sherman NE, Jeffery ED, et al. Accurate label-free protein quantitation with high- and low-resolution mass spectrometers. J Proteome Res. 2014;13(2):1034–44. doi: 10.1021/pr401017h 24295401 PMC3946283

[69] Bartholomaus A, Fedyunin I, Feist P, Sin C, Zhang G, Valleriani A, et al. Bacteria differently regulate mRNA abundance to specifically respond to various stresses. Philos Trans A Math Phys Eng Sci. 2016;374(2063). doi: 10.1098/rsta.2015.0069 26857681

[70] Segura A, Bertoni M, Auffret P, Klopp C, Bouchez O, Genthon C, et al. Transcriptomic analysis reveals specific metabolic pathways of enterohemorrhagic *Escherichia coli* O157:H7 in bovine digestive contents. BMC Genomics. 2018;19(1):766. doi: 10.1186/s12864-018-5167-y 30352567 PMC6199705

[71] Bertin Y, Segura A, Jubelin G, Duniere L, Durand A, Forano E. Aspartate metabolism is involved in the maintenance of enterohaemorrhagic *Escherichia coli* O157:H7 in bovine intestinal content. Environ Microbiol. 2018;20(12):4473–85. doi: 10.1111/1462-2920.14380 30109758

[72] Nguyen YN, Sheng H, Dakarapu R, Falck JR, Hovde CJ, Sperandio V. The acyl-homoserine lactone synthase YenI from *Yersinia enterocolitica* modulates virulence gene expression in enterohemorrhagic *Escherichia coli* O157:H7. Infect Immun. 2013;81(11):4192–9. doi: 10.1128/IAI.00889-13 23980115 PMC3811827

[73] Tsai MF, McCarthy P, Miller C. Substrate selectivity in glutamate-dependent acid resistance in enteric bacteria. Proc Natl Acad Sci U S A. 2013;110(15):5898–902. doi: 10.1073/pnas.1301444110 23530225 PMC3625338

[74] Tsai MF, Miller C. Substrate selectivity in arginine-dependent acid resistance in enteric bacteria. Proc Natl Acad Sci U S A. 2013;110(15):5893–7. doi: 10.1073/pnas.1301442110 23530220 PMC3625328

[75] Foster JW. *Escherichia coli* acid resistance: tales of an amateur acidophile. Nat Rev Microbiol. 2004;2(11):898–907. doi: 10.1038/nrmicro1021 15494746

