# Supplementary figures and images for: Novel reusable animal model for comparative evaluation of in vivo growth and protein-expression of Escherichia coli O157 strains in the bovine rumen
Source: PLoS One. 2022 May 26;17(5):e0268645. doi: 10.1371/journal.pone.0268645 (PMC9135228; doi:10.1371/journal.pone.0268645)

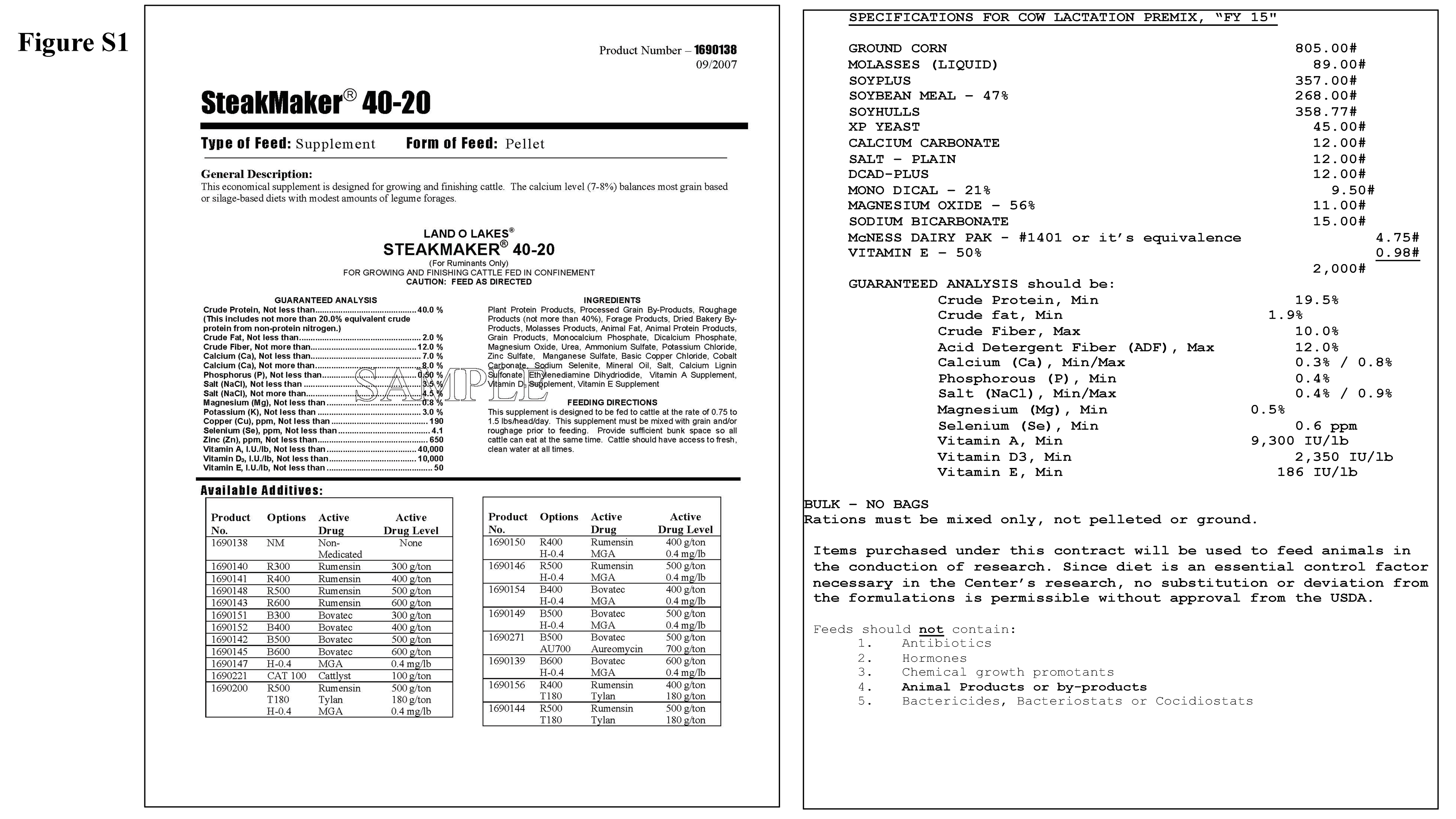

Supplement: S1 Fig — (TIF) [file pone.0268645.s001.tif]

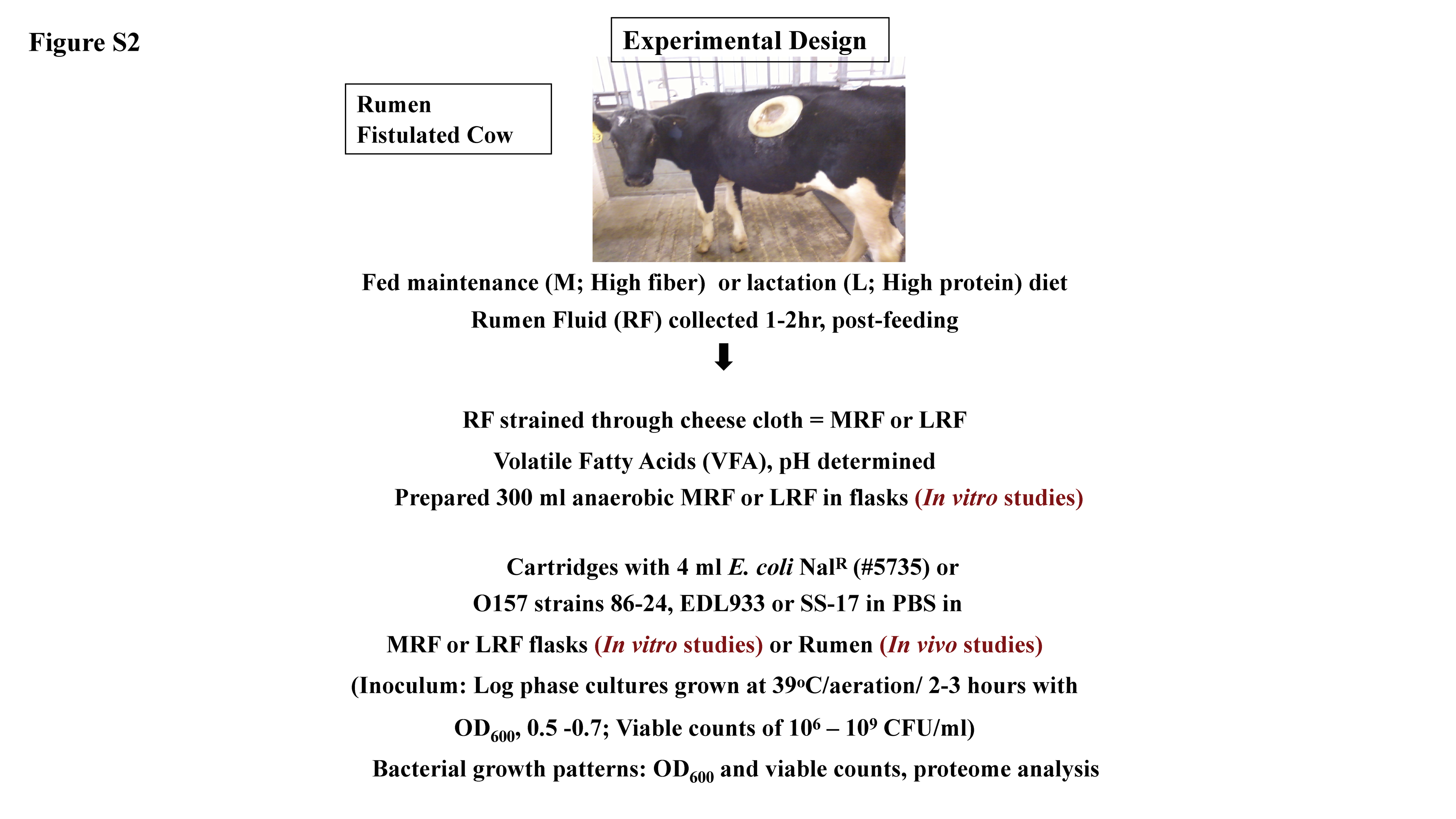

Supplement: S2 Fig — (TIF) [file pone.0268645.s002.tif]

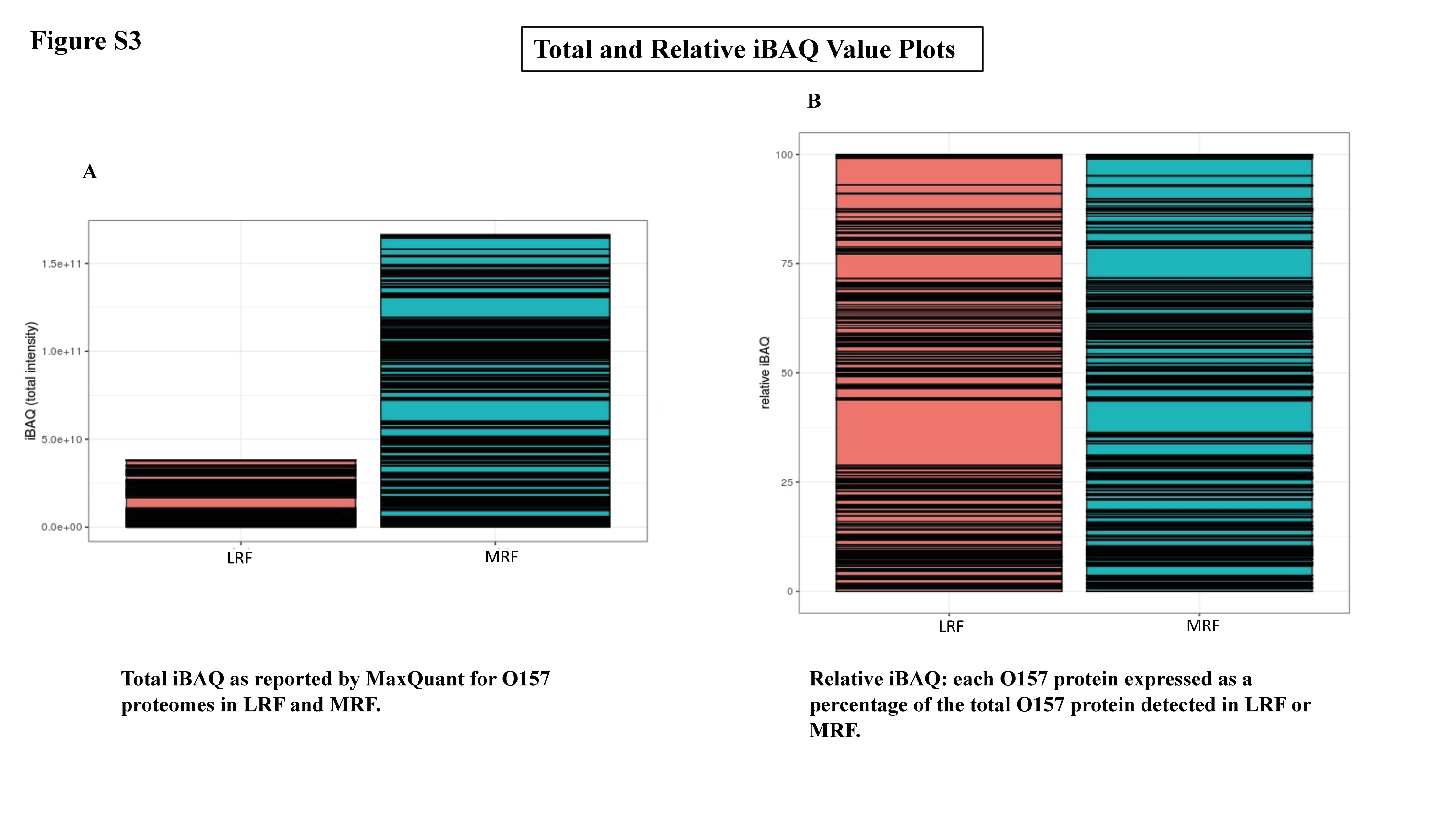

Supplement: S3 Fig — (TIF) [file pone.0268645.s003.tif]

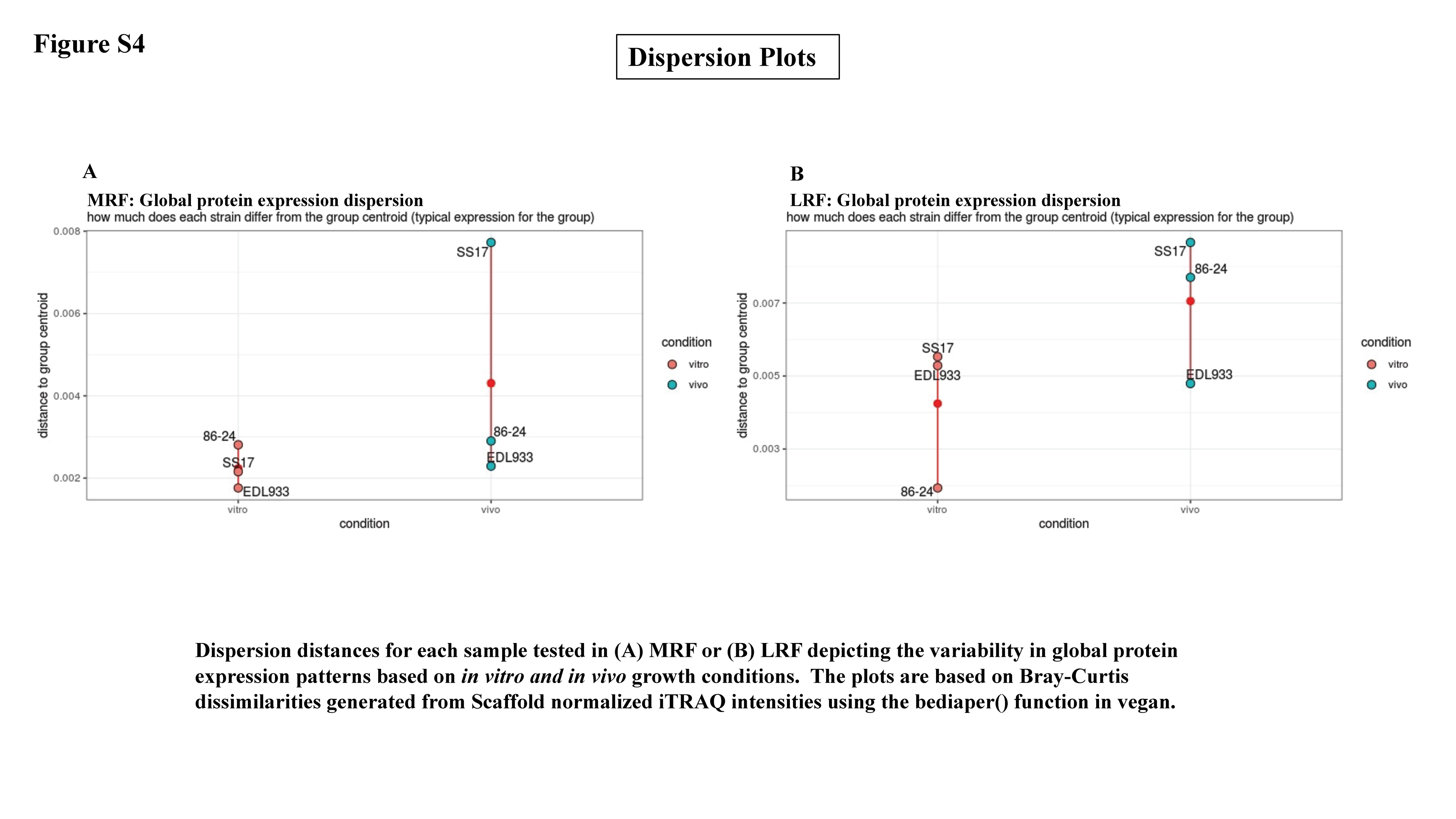

Supplement: S4 Fig — (TIF) [file pone.0268645.s004.tif]
