# Supplementary material for: Novel reusable animal model for comparative evaluation of in vivo growth and protein-expression of Escherichia coli O157 strains in the bovine rumen
Source: PLoS One. 2022 May 26;17(5):e0268645. doi: 10.1371/journal.pone.0268645 (PMC9135228; doi:10.1371/journal.pone.0268645)
Supplement: S1 Table — (DOCX) [file pone.0268645.s005.docx]

**Table S1. Recovery of *E. coli* Nal^R^ (#5735) in dialysis PVDF tubings versus cartridges exposed to MRF**

**in *in vitro* and *in vivo* pilot experiments.**

| **Dialysis system**  **/Sampling time** | | ***In vitro* in Rumen fluid^1^ from**  **Animal #A** | | ***In vivo* in the Rumen of**  **Animal #A** | |
| --- | --- | --- | --- | --- | --- |
|  |  | **Average^2^**  **Bacterial counts**  **(cfu/ml)** | **Numbers/Volume^3^** | **Average Bacterial counts**  **(cfu/ml)** | **Numbers/Volume** |
| **PVDF tubing** | **0 h** | 6 ± 1 x 10^7^ | 2 tubings/4 ml each | 2.7 ± 0.7 x 10^9^ | 2 tubings/4 ml each |
|  | **48 h** | 3 x 10^7^ | 2 tubings/2 ml each | 2.9 x 10^7^ | 1 tubing/200 µl |
| **Cartridge** | **0 h** | 6 ± 1 x 10^7^ | 2 cartridges/4 ml each | 6.1 ± 5 x 10^8^ | 2 cartridges/4 ml each |
|  | **48 h** | 3 ± 1 x 10^7^ | 2 cartridges/3 ml each | 2.4 + 1.7x 10^7^ | 2 cartridges/3 ml each |

^1^Incubated in flasks at 39^o^C with shaking under anaerobic conditions.

^2^Average of counts from 2 tubings or cartridges when available.

^3^Number of tubings or cartridges and volume of bacterial suspension within, at the beginning (0 h) and end (48 h) of the experiment.
