# Supplementary material for: Novel reusable animal model for comparative evaluation of in vivo growth and protein-expression of Escherichia coli O157 strains in the bovine rumen
Source: PLoS One. 2022 May 26;17(5):e0268645. doi: 10.1371/journal.pone.0268645 (PMC9135228; doi:10.1371/journal.pone.0268645)
Supplement: S2 Table — (DOCX) [file pone.0268645.s006.docx]

**Table S2. Recovery of bacteria from cartridges exposed to MRF in *in vitro* and *in vivo*.**

| **Bacterial strain** | | ***In vitro* in MRF** | ***In vivo* in MRF** |
| --- | --- | --- | --- |
|  |  | **Average^1^ Bacterial counts (cfu/ml)** | **Average Bacterial counts (cfu/ml)** |
| **O157 strain 86-24** | **0 h** | 9.3 ± 7 x 10^6^ | 1.8 ± 0.4 x 10^9^ |
|  | **48 h** | 1.6 ± 0.4 x 10^4^ | 4.4 ± 4 x 10^8^ |
| **O157 strain EDL933** | **0 h** | 8 ± 7 x 10^6^ | 1.6 ± 0.9 x 10^9^ |
|  | **48 h** | 3.5 ± 0.5 x 10^4^ | 2.7 ± 2.5 x 10^8^ |
| **O157 strain SS-17** | **0 h** | 1.2 ± 0.5 x 10^8^ | 4 ± 3 x 10^9^ |
|  | **48 h** | 2.2 ± 0.8 x 10^7^ | 8 ± 6 x 10^7^ |
| ***E. coli* Nal^R^ (#5735)** | **0 h** | 3.9 ± 3.8 x 10^8^ | 6 ± 4 x 10^8^ |
|  | **48 h** | 1.9 ± 0.1 x 10^5^ | 2.4 ± 1.2 x 10^7^ |

^1^Average from two separate experiments.
