# Supplementary material for: Novel reusable animal model for comparative evaluation of in vivo growth and protein-expression of Escherichia coli O157 strains in the bovine rumen
Source: PLoS One. 2022 May 26;17(5):e0268645. doi: 10.1371/journal.pone.0268645 (PMC9135228; doi:10.1371/journal.pone.0268645)
Supplement: S3 Table — (DOCX) [file pone.0268645.s007.docx]

**Table S3. Recovery of bacteria from cartridges exposed to LRF in *in vitro* and *in vivo*.**

| **Bacterial strain** | | ***In vitro* in LRF** | ***In vivo* in LRF** |
| --- | --- | --- | --- |
|  |  | **Average^1^ Bacterial counts (cfu/ml)** | **Average Bacterial counts (cfu/ml)** |
| **O157 strain 86-24** | **0 h** | 4.4 ± 4 x 10^7^ | 2 ± 1.5 x 10^9^ |
|  | **48 h** | 7 ± 6 x 10^5^ | 1 ± 0.9 x 10^7^ |
| **O157 strain EDL933** | **0 h** | 1 ± 0.7 x 10^8^ | 5 ± 4 x 10^8^ |
|  | **48 h** | 2 ± 2 x 10^6^ | 5 ± 5 x 10^6^ |
| **O157 strain SS-17** | **0 h** | 1.2 ± 0.5 x 10^8^ | 1.5 ± 1.3 x 10^9^ |
|  | **48 h** | 1.6 ± 1.6 x 10^3^ | 2.9 ± 2.8 x 10^7^ |
| ***E. coli* Nal^R^ (#5735)** | **0 h** | 9 ± 7 x 10^6^ | 7.5 ± 6.5 x 10^8^ |
|  | **48 h** | 2 ± 2 x 10^5^ | 1 ± 0.9 x 10^5^ |

^1^Average from two separate experiments.
