# Supplementary material for: Novel reusable animal model for comparative evaluation of in vivo growth and protein-expression of Escherichia coli O157 strains in the bovine rumen
Source: PLoS One. 2022 May 26;17(5):e0268645. doi: 10.1371/journal.pone.0268645 (PMC9135228; doi:10.1371/journal.pone.0268645)
Supplement: S1 Text — (PDF) [file pone.0268645.s014.pdf]

Parameter	Value
Version	1.6.7.0
User name	julian.trachsel
Machine name	arsiaam0nfsep11
Date of writing	08/04/2019 17:06:38
Include contaminants	True
PSM FDR	0.01
PSM FDR Crosslink	0.01
Protein FDR	0.01
Site FDR	0.01
Use Normalized Ratios For Occupancy	True
Min. peptide Length	7
Min. score for unmodified peptides	0
Min. score for modified peptides	40
Min. delta score for unmodified peptides	0
Min. delta score for modified peptides	6
Min. unique peptides	0
Min. razor peptides	1
Min. peptides	1
Use only unmodified peptides and	True
Modifications included in protein quantification	Oxidation (M);Acetyl (Protein N-term)
Peptides used for protein quantification	Razor
Discard unmodified counterpart peptides	True
Label min. ratio count	2
Use delta score	False
iBAQ	True
iBAQ log fit	True
Match between runs	False
Find dependent peptides	False
Fasta file	/home/julian.trachsel/Indira/reference/NCBI_O157_clustered_99.faa;/home/julian.trachsel/Indira/reference/UP000000625_ecoli_pan_proteome.fasta
Decoy mode	revert
Include contaminants	True
Advanced ratios	True
Fixed andromeda index folder	
Temporary folder	
Combined folder location	
Second peptides	True
Stabilize large LFQ ratios	True
Separate LFQ in parameter groups	True
Require MS/MS for LFQ comparisons	True
Calculate peak properties	False
Main search max. combinations	200
Advanced site intensities	True
Write msScans table	False
Write msmsScans table	True
Write ms3Scans table	True
Write allPeptides table	True

Write mzRange table	True
Write pasefMsmsScans table	True
Write accumulatedPasefMsmsScans table	True
Max. peptide mass [Da]	4600
Min. peptide length for unspecific search	8
Max. peptide length for unspecific search	25
Razor protein FDR	True
Disable MD5	False
Max mods in site table	3
Match unidentified features	False
Epsilon score for mutations
Evaluate variant peptides separately	True
Variation mode	None
MS/MS tol. (FTMS)	20 ppm
Top MS/MS peaks per Da interval. (FTMS)	12
Da interval. (FTMS)	100
MS/MS deisotoping (FTMS)	True
MS/MS deisotoping tolerance (FTMS)	7
MS/MS deisotoping tolerance unit (FTMS)	ppm
MS/MS higher charges (FTMS)	True
MS/MS water loss (FTMS)	True
MS/MS ammonia loss (FTMS)	True
MS/MS dependent losses (FTMS)	True
MS/MS recalibration (FTMS)	False
MS/MS tol. (ITMS)	0.5 Da
Top MS/MS peaks per Da interval. (ITMS)	8
Da interval. (ITMS)	100
MS/MS deisotoping (ITMS)	False
MS/MS deisotoping tolerance (ITMS)	0.15
MS/MS deisotoping tolerance unit (ITMS)	Da
MS/MS higher charges (ITMS)	True
MS/MS water loss (ITMS)	True
MS/MS ammonia loss (ITMS)	True
MS/MS dependent losses (ITMS)	True
MS/MS recalibration (ITMS)	False
MS/MS tol. (TOF)	40 ppm
Top MS/MS peaks per Da interval. (TOF)	10
Da interval. (TOF)	100
MS/MS deisotoping (TOF)	True
MS/MS deisotoping tolerance (TOF)	0.01
MS/MS deisotoping tolerance unit (TOF)	Da
MS/MS higher charges (TOF)	True
MS/MS water loss (TOF)	True
MS/MS ammonia loss (TOF)	True
MS/MS dependent losses (TOF)	True
MS/MS recalibration (TOF)	False
MS/MS tol. (Unknown)	20 ppm
Top MS/MS peaks per Da interval. (Unknown)	12
Da interval. (Unknown)	100
MS/MS deisotoping (Unknown)	True

MS/MS deisotoping tolerance (Unknown)	7
MS/MS deisotoping tolerance unit (Unknown)	ppm
MS/MS higher charges (Unknown)	True
MS/MS water loss (Unknown)	True
MS/MS ammonia loss (Unknown)	True
MS/MS dependent losses (Unknown)	True
MS/MS recalibration (Unknown)	False
Site tables	Oxidation (M)Sites.txt
