# Supplementary material for: Novel reusable animal model for comparative evaluation of in vivo growth and protein-expression of Escherichia coli O157 strains in the bovine rumen
Source: PLoS One. 2022 May 26;17(5):e0268645. doi: 10.1371/journal.pone.0268645 (PMC9135228; doi:10.1371/journal.pone.0268645)
Supplement: S2 Text — (PDF) [file pone.0268645.s015.pdf]

GCA_000006665.1_ASM666v1
GCA_000008865.2_ASM886v2
GCA_000021125.1_ASM2112v1
GCA_000022225.1_ASM2222v1
GCA_000155125.1_ASM15512v1
GCA_000171915.1_ASM17191v1
GCA_000171935.1_ASM17193v1
GCA_000171955.1_ASM17195v1
GCA_000171975.1_ASM17197v1
GCA_000171995.1_ASM17199v1
GCA_000172015.1_ASM17201v1
GCA_000172035.1_ASM17203v1
GCA_000172055.1_ASM17205v1
GCA_000181735.1_ASM18173v1
GCA_000181755.1_ASM18175v1
GCA_000181775.1_ASM18177v1
GCA_000187285.4_ASM18728v4
GCA_000187305.2_ASM18730v2
GCA_000187325.2_ASM18732v2
GCA_000187385.2_ASM18738v2
GCA_000192665.2_ASM19266v2
GCA_000192685.2_ASM19268v2
GCA_000193955.2_ASM19395v2
GCA_000247665.4_EcoliT22_2.0
GCA_000267425.2_ASM26742v2
GCA_000614085.2_Ec2011EL-2288
GCA_000614255.1_Ec2011EL-2114
GCA_000614305.2_Ec2011EL-2286
GCA_000614345.2_Ec2011EL-2312
GCA_000614405.2_Ec2011EL-2287
GCA_000614645.2_Ec2011EL-2113
GCA_000614685.2_Ec2011EL-2289
GCA_000614805.2_EcF7350
GCA_000614885.2_Ec2010C-4979C1
GCA_000614965.1_Ec2009EL1705
GCA_000615015.1_Ec2009EL2109
GCA_000615695.1_Ec2011EL-2101
GCA_000615745.2_Ec2011EL-2103
GCA_000615785.2_Ec2011EL-2104
GCA_000615815.2_Ec2011EL-2105
GCA_000615865.2_Ec2011EL-2106
GCA_000615925.2_Ec2011EL-2107
GCA_000615965.2_Ec2011EL-2108
GCA_000616005.2_Ec2011EL-2109
GCA_000616035.2_Ec2011EL-2111
GCA_000616075.2_Ec2011EL-2112
GCA_000616365.1_Ec2011EL-1107
GCA_000616405.2_Ec2011EL-2090
GCA_000616425.2_Ec2011EL-2091
GCA_000616445.2_Ec2011EL-2092

GCA_000616465.2_Ec2011EL-2093
GCA_000616485.2_Ec2011EL-2094
GCA_000616505.2_Ec2011EL-2096
GCA_000616525.2_Ec2011EL-2097
GCA_000616545.2_Ec2011EL-2098
GCA_000616585.2_Ec08-3037
GCA_000616605.2_Ec08-3527
GCA_000616645.2_Ec08-4169
GCA_000616705.2_Ec2011EL-2099
GCA_000616745.1_EcK5806
GCA_000617445.2_Ec06-3745
GCA_000617665.2_Ec07-3091
GCA_000617705.2_Ec06-4039
GCA_000617725.2_Ec07-3391
GCA_000617965.1_EcK4405
GCA_000618005.2_Ec98-3133
GCA_000618025.1_EcK1420
GCA_000618045.1_EcF6749
GCA_000618065.2_EcF7384
GCA_000618105.2_EcF6142
GCA_000618145.2_EcF7410
GCA_000618185.1_EcG5303
GCA_000618225.2_EcF7377
GCA_000618245.1_EcH2495
GCA_000618265.2_EcF6751
GCA_000618285.2_EcH2498
GCA_000618305.2_EcF6750
GCA_000618325.1_EcK1796
GCA_000618345.1_EcK1845
GCA_000618365.1_EcK1793
GCA_000618385.1_EcK1795
GCA_000618425.1_EcK1792
GCA_000618445.1_EcK1921
GCA_000618465.1_EcK1927
GCA_000618485.1_EcK2188
GCA_000618505.1_EcK2191
GCA_000618525.1_EcK2581
GCA_000618545.1_EcK2622
GCA_000618565.1_EcK2845
GCA_000618585.1_EcK2854
GCA_000618605.1_EcK4396
GCA_000618625.1_EcK4406
GCA_000618645.1_EcK2192
GCA_000618665.1_EcK2324
GCA_000618685.1_EcK4527
GCA_000618745.1_EcK5418
GCA_000618765.1_EcK5448
GCA_000618785.1_EcK5449
GCA_000618805.1_EcK5453
GCA_000618825.1_EcK5467

GCA_000618845.1_EcK5602
GCA_000618865.1_EcK5607
GCA_000618885.1_EcK5460
GCA_000618925.2_EcK7140
GCA_000618945.2_Ec2011EL-2290
GCA_000618985.2_Ec2011EL-2313
GCA_000619045.2_Ec2009C-4258
GCA_000619145.1_Ec2009EL1449
GCA_000619165.1_Ec2009EL1913
GCA_000619365.1_EcK5852
GCA_000619385.1_EcK6590
GCA_000619405.1_EcK6676
GCA_000619425.1_EcK6687
GCA_000619445.1_EcK5609
GCA_000619665.1_Ec08-4529
GCA_000619685.2_Ec08-4540
GCA_000632555.1_Ec2010EL-2044
GCA_000632655.1_Ec2010EL-2045
GCA_000730345.1_ASM73034v1
GCA_000732965.1_ASM73296v1
GCA_000803705.1_ASM80370v1
GCA_000835045.1_ASM83504v1
GCA_000835055.1_734_3
GCA_000978815.1_ASM97881v1
GCA_000978845.1_ASM97884v1
GCA_001006425.1_ASM100642v1
GCA_001307215.1_ASM130721v1
GCA_001440045.1_ASM144004v1
GCA_001516935.2_ASM151693v2
GCA_001558995.2_ASM155899v2
GCA_001651925.2_ASM165192v2
GCA_001651945.2_ASM165194v2
GCA_001651965.2_ASM165196v2
GCA_001695515.1_ASM169551v1
GCA_001753445.1_ASM175344v1
GCA_001753465.1_ASM175346v1
GCA_001753485.1_ASM175348v1
GCA_001753505.1_ASM175350v1
GCA_001753525.1_ASM175352v1
GCA_001753545.1_ASM175354v1
GCA_001753565.1_ASM175356v1
GCA_001953095.1_ASM195309v1
GCA_002027605.1_ASM202760v1
GCA_002027645.1_ASM202764v1
GCA_002027685.1_ASM202768v1
GCA_002206405.2_ASM220640v2
GCA_002208865.2_ASM220886v2
GCA_002259555.1_ASM225955v1
GCA_002806745.1_ASM280674v1
GCA_002806795.1_ASM280679v1

GCA_003194645.1_ASM319464v1
GCA_003194655.1_ASM319465v1
GCA_003719795.1_ASM371979v1
GCA_003722195.1_ASM372219v1
GCA_003966795.1_ASM396679v1
GCA_004100305.1_ASM410030v1
GCA_004118915.1_ASM411891v1
GCA_004137365.1_ASM413736v1
GCA_005037735.2_ASM503773v2
GCA_005885915.1_ASM588591v1
GCA_005885955.1_ASM588595v1
GCA_006514375.1_ASM651437v1
